# Compound A, a Selective Glucocorticoid Receptor Modulator, Enhances Heat Shock Protein Hsp70 Gene Promoter Activation

**DOI:** 10.1371/journal.pone.0069115

**Published:** 2013-07-30

**Authors:** Ilse M. Beck, Zuzanna J. Drebert, Ruben Hoya-Arias, Ali A. Bahar, Michael Devos, Dorien Clarisse, Sofie Desmet, Nadia Bougarne, Bart Ruttens, Valerie Gossye, Geertrui Denecker, Sam Lievens, Marc Bracke, Jan Tavernier, Wim Declercq, Kris Gevaert, Wim Vanden Berghe, Guy Haegeman, Karolien De Bosscher

**Affiliations:** 1 Laboratory of Experimental Cancer Research (LECR), Department of Radiation Therapy & Experimental Cancer Research, Ghent University, Ghent, Belgium; 2 Human Oncology & Pathogenesis Program, Memorial Sloan-Kettering Cancer Center, New York, New York, United States of America; 3 Cytokine Receptor Lab, Department of Medical Protein Research, VIB, Ghent, Belgium; 4 Cytokine Receptor Lab, Department of Biochemistry, Ghent University, Ghent, Belgium; 5 Molecular Signaling and Cell Death Unit, VIB Department for Molecular Biomedical Research, Ghent University, Ghent (Zwijnaarde), Belgium; 6 Lab Protein Chemistry, Proteomics & Epigenetic Signaling (PPES), Department Biomedical Sciences, University of Antwerp, Wilrijk, Belgium; 7 Department of Medical Protein Research, VIB, Ghent, Belgium; 8 Department of Biochemistry, Ghent University, Ghent, Belgium; Boston University Medical School, United States of America

## Abstract

Compound A possesses glucocorticoid receptor (GR)-dependent anti-inflammatory properties. Just like classical GR ligands, Compound A can repress NF-κB-mediated gene expression. However, the monomeric Compound A-activated GR is unable to trigger glucocorticoid response element-regulated gene expression. The heat shock response potently activates heat shock factor 1 (HSF1), upregulates Hsp70, a known GR chaperone, and also modulates various aspects of inflammation. We found that the selective GR modulator Compound A and heat shock trigger similar cellular effects in A549 lung epithelial cells. With regard to their anti-inflammatory mechanism, heat shock and Compound A are both able to reduce TNF-stimulated IκBα degradation and NF-κB p65 nuclear translocation. We established an interaction between Compound A-activated GR and Hsp70, but remarkably, although the presence of the Hsp70 chaperone as such appears pivotal for the Compound A-mediated inflammatory gene repression, subsequent novel Hsp70 protein synthesis is uncoupled from an observed CpdA-induced Hsp70 mRNA upregulation and hence obsolete in mediating CpdA’s anti-inflammatory effect. The lack of a Compound A-induced increase in Hsp70 protein levels in A549 cells is not mediated by a rapid proteasomal degradation of Hsp70 or by a Compound A-induced general block on translation. Similar to heat shock, Compound A can upregulate transcription of Hsp70 genes in various cell lines and BALB/c mice. Interestingly, whereas Compound A-dependent Hsp70 promoter activation is GR-dependent but HSF1-independent, heat shock-induced Hsp70 expression alternatively occurs in a GR-independent and HSF1-dependent manner in A549 lung epithelial cells.

## Introduction

Inflammation is a complex immune response of tissues to harmful stimuli, such as the self-produced tumor necrosis factor (TNF) characterized by an activator protein-1 (AP-1) and/or nuclear factor κB (NF-κB)-mediated generation of cytokines and chemokines, e.g. IL6 and IL8. In unstimulated cells, NF-κB is restrained in the cytoplasm by the NF-κB-binding inhibitory protein IκB. Upon induction, the IκB kinase (IKK) complex, comprising two catalytic components, IKKα and IKKβ, and a regulatory component IKKγ (NEMO), can phosphorylate IκB, destining this inhibitory factor for ubiquitination and subsequent degradation by the 26S proteasome. Ensuing, the freed and activated NF-κB p65-p50 heterodimer translocates into the nucleus, where it will bind onto specific promoter recognition sites and activate multiple pro-inflammatory genes [1].

Glucocorticoids (GCs), the steroidal ligands of the glucocorticoid receptor (GR, NR3C1), are efficaciously used as anti-inflammatory drugs. Structurally, the GR comprises a N-terminal domain in which a transactivation function is encoded, a DNA-binding domain which also functions in GR dimerization, and a C-terminal ligand-binding domain which harbors a second transactivation function [2]. The unactivated GR resides predominantly in the cytoplasm where a chaperone complex consisting of heat shock protein 70 (Hsp70), Hsp90 and variable immunophilins keeps the receptor in a ligand-receptive state [3]. After GCs bind to the GR, the receptor’s conformation changes and as a result GR sheds its chaperone complex and translocates into the nucleus, where it can activate or repress specific gene transcription [2]. The GC-activated GR can positively affect gene expression via dimerized GR binding onto specific GC-responsive elements (GREs) in the promoter. Conversely, GC-activated GR can negatively interfere with gene expression via diverse mechanisms among which the binding of GR to a negative GRE, tethering of monomer GR to transcription factors such as NF-κB, and the alteration of the composition of the transcription-initiating enhanceosome [2]. However, long-term therapy with GCs is burdened with a detrimental side-effect profile [4] driving ongoing research to develop new therapeutic strategies to combat inflammation. With this aim, we investigate Compound A (CpdA), a phenyl aziridine precursor molecule established as a selective GR modulator. CpdA is able to alter GR’s conformation and drives it into the nucleus [5]. However, unlike classical GCs [6], CpdA does not stimulate GR Ser^211^ phosphorylation [5] or GR dimerization [7]. Hence, CpdA-modulated GR does not transactivate GRE-regulated gene expression and safeguards the system from various classic GC-associated side effects [5]. This selective GR modulator is, however, a potent repressor of NF-κB-driven pro-inflammatory gene expression, both *in vitro* and *in vivo* [8]. Mechanistically, CpdA-activated GR diminishes the TNF-stimulated NF-κB transactivation capacity and DNA binding [5]. As most GC therapy-associated side effects are linked to GR transactivation mechanisms [9], a selective GR modulator possibly holds great promise in future therapeutics combating inflammation.

Various stressors, including heat and proteotoxic stress, instigate the heat shock response [10], [11]. This reaction functions to protect the cell and is characterized by the activation of the transcription factor heat shock factor 1 (HSF1) followed by the elevated gene expression of inducible heat shock proteins (Hsps), such as Hsp70 [11]. The heat shock-activated mechanism to stimulate Hsp70 promoter activity occurs via the activation of the transcription factor HSF1. In a resting state, HSF1 is distributed throughout the cell as a monomer. Upon activation, HSF1 becomes hyperphosphorylated, trimerizes and aggregates in nuclear stress granules [12], [13]. The activated HSF1 oligomers can bind onto a specific heat shock response element (HSE) to activate gene transcription of various inducible Hsps [14], [15].

Physiologically, Hsp70 can play a role as a chaperoning molecule, ensuring proper folding and refolding of various proteins. As such, Hsp70 contributes to keep the unactivated GR in a ligand-receptive state [3]. The presence of Hsp70 has also been shown to be crucial for mice to resist TNF-induced lethality [16]. Moreover, elevated intracellular Hsp70 and activated HSF1 have been described to have anti-inflammatory abilities via disruption of the IKK complex, abrogation of IκBα degradation and consecutive NF-κB p65 translocation [17], [18].

In brief, we report here on the modulation of Hsp70 promoter activity via different stimuli. Interestingly, the SGRM CpdA upregulates Hsp70 mRNA via a GR-dependent and HSF1-independent mechanism. Although, CpdA, similar to heat shock, hampers TNF-stimulated IκBα degradation and NF-κB p65 nuclear translocation, its anti-inflammatory mechanism does not seem to require absolute de novo protein synthesis. Nonetheless, the presence of adequate levels of the Hsp70 chaperone is absolutely necessary to allow CpdA to repress NF-κB-driven gene expression.

## Materials and Methods

### Cell Culture & Reagents

A549 adenocarcinoma human alveolar basal epithelial cells were a kind gift from Dr. Ian Adcock [19]. PC-3 prostate adenocarcinoma cells were a kind gift from Dr. Schalken and Dr. Giroldi (Nijmegen, The Netherlands) [20]. HEK293T cells (ATCC® CRL-11268™), L929sA murine fibrosarcoma cells [21] and other cells were cultured in DMEM with 10% fetal calf serum, 100U/ml penicillin and 0.1mg/ml streptomycin. All cell lines were grown at 37°C under 5% CO_2_.

Recombinant TNFα production and purification to 99% homogeneity has been described previously [22]. Dexamethasone (DEX) and cycloheximide (CHX) were obtained from Sigma-Aldrich. CpdA was produced and stored as described [5]. TNFα was used at a final concentration of 2000IU/ml and dissolved in medium. DEX and CpdA were dissolved in ethanol and used, respectively, at a 1µM and 10µM final concentration, unless stated otherwise in the figure legend. Cycloheximide (CHX) was dissolved in DMSO and used in a final concentration of 20µg/ml.

### Animals and Ethics Statement

BALB/c mice were obtained from Charles River. The animal experiment was performed in accordance with all Belgian legal and ethical requirements and approved by the local ethics committee of Ghent University (Permit number: 07–012). In order to minimize suffering, intraperitoneal injections were performed with 26G needles and mice were sacrificed by cervical dislocation under Ketamin/Xylazin anaesthesia.

### Plasmids

The plasmid p(IL6κB)_3_50hu.IL6P-luc+ and p(GRE)_2_50hu.IL6P-luc+ were described previously [23], [24]. The p(GRE)_2_50hu.IL6P-luc+ plasmid was derived from the p(IL6κB)_3_50hu.IL6P-luc+ by replacing the κB motifs with two consensus GRE sites. The heat-inducible mHsp70i-luc reporter gene construct was kindly donated to us by Dr. H Moo Kwon (Baltimore, Maryland) [25].

### Bait, prey and Reporter Constructs

All constructs used in this report were generated by standard PCR- or restriction based cloning procedures. The pCLG-GR plasmid was generated by amplifying the human GR-coding sequence of a hGR-containing expression plasmid using primers GGGGAGCTCCGACTCCAAAGAATCATTAAC and GGGGCGGCCGCTCACTTTTGATGAAACAGAA, cutting the amplicon with SacI and NotI restriction enzymes and ligating the resulting fragment into SacI-NotI digested pCLG backbone [26]. The pMG1 plasmid encoding an unfused gp130 receptor fragment as an empty prey control was obtained by opening the pMG1 vector [27] using EcoRI and XhoI, blunting the vector backbone through Pfu DNA Polymerase and self-ligation. The prey plasmids pMG1-HSP90AA1 and pMG1-HSP70 were created by Gateway transfer of the full size HSP90AA1 and HSP70 ORFs, obtained as entry clones from the hORFeome collection [28] into the Gateway compatible pMG1 prey destination vector as described earlier [29]. The pXP2d2-rPAP1-luciferase reporter has been described elsewhere [30], [31].

### Transfection & Reporter Gene Assays

Stable transfection of mHsp70i-luc into L929sA cells was performed by the calcium phosphate precipitation protocol as described [23]. Transient transfections of p(IL6κB)_3_50hu.IL6P-luc+ or p(GRE)_2_50hu.IL6P-luc+ in A549 cells were performed via lipofectamine (Invitrogen)-based procedures, as described [32]. Post-inductions, cells were washed with PBS and lysed (TROPIX). Total solvent concentration was kept similar in all conditions. Reporter gene expression was corrected by normalization to the co-expressed β-galactosidase (β-gal) protein levels, as assayed via a Galacto-Light kit (TROPIX).

For the MAPPIT analysis, HEK293T cells were cultured in a 8% CO_2_ humidified atmosphere at 37°C and grown in DMEM with 10% fetal calf serum (Gibco®). 10 000 cells were seeded in black 96-well plates. One day later, the cells were transiently co-transfected with either a DHFR-bait plasmid (irrelevant bait, as a negative control) or a GRα-bait plasmid, together with the STAT3-responsive rPAP1-luci reporter plasmid and the desired prey-plasmid. The empty prey was used as a negative control. Twenty-four hours after transfection cells were stimulated with leptin (100ng/ml) or leptin in combination with DEX (1µM) or CpdA (10µM) for another 24h or were left untreated (NS). All inductions were performed at least in triplicate. Total cell lysates were assayed for promoter activity of the relative reporter genes via a luciferase assay, performed according to manufacturer’s instructions (Promega). Luciferase activity was measured by chemiluminescence in a TopCount NXT luminometer (Perkin Elmer).

### RT-(q)PCR

For inductions, total solvent concentration was kept similar in all conditions. Total RNA was isolated using TRIzol Reagent (Life Technologies). cDNA was generated with minimally 500ng of total RNA, using oligo-d(T) primers and MMLV RT enzyme (Promega) and amplified with Taq polymerase (Promega) and sequence-specific primers targeted at HSPA1A, HPSA1B, or GAPDH PCR products, generated under non-saturating conditions. The products were separated by PAGE and were detected by ethidium bromide staining and subsequently visualized using UV. To quantify the bands obtained via RT-PCR, we applied ImageJ software based analysis (http://rsb.info.nih.gov/ij/). The area under curve (AUC) of the specific signal was corrected for the AUC of the housekeeping gene control.

Female 8 week old BALB/c mice were subjected to the indicated treatment and total RNA was purified out of total skin grafts using RNeasy+ kit (Qiagen). RNA was transcribed to cDNA with iScript cDNA synthesis kit (Bio-Rad). RT-qPCR analysis of animal and cellular samples was performed using the Lightcycler 480 system and Lightcycler qPCR reagents (Roche), to assay specific GR, HSPA1A, HSPA1B, IL8, GAPDH, β-actin, cyclophilin, 36B4, 28S, RPL13a, HMBS, ACTB mRNA levels. qPCR was performed in triplicate. All primer sequences are available upon request. Specific signal was normalized to housekeeping control.

### Protein Analyses & Antibodies

For Western analysis, total cell lysates were prepared using SDS sample buffer (50mM Tris pH6.8; 2% SDS; 10% glycerol; bromophenol blue, 100mM DTT) or TOTEX buffer (20 mM Hepes/KOH pH 7.9; 0.35 M NaCl; 20% glycerol; 1% NP40; 1 mM MgCl2; 0.5 mM EDTA; 0.1 mM EGTA; 2mM pefabloc; 10µg/ml aprotinin; 5mM DTT) followed by standard Western blotting and antibody probing procedures (Santa Cruz Biotechnology). Samples prepared with SDS sample buffer are loaded at an equal volume of maximally 30µl and equal loading is assayed via a loading control. Samples prepared with TOTEX buffer are analysed for protein concentration via a Bradford analysis [33] and maximally 40µg is loaded for Western blot analysis.

The antibodies (Abs), used in the Western blot analyses, were directed against GR, PARP, NF-κB p65, IκBα and tubulin (Santa Cruz Biotechnology), β-catenin (Sigma-Aldrich) and phosphorylated S6 ribosomal protein (Cell Signalling Technology). Antibodies directed against inducible Hsp70 were purchased via different companies, but it was verified that all recognize inducible Hsp70: anti-Hsp72 SPA-810 monoclonal Ab (Stressgen) and anti-Hsp70 polyconal Ab(Santa Cruz Biotechnologies; sc-1060). All primary antibodies were used at 1/1000 dilutions; except anti-tubulin, which was used at 1/2000. The secondary antibodies were used with the following dilutions: anti-Mouse IgG-HRP (NA931V, GE-Healthcare UK Limited) at 1/3000, anti-Rabbit IgG-HRP (NA935V, GE-Healthcare UK Limited) at 1/4000, anti-Rat IgG-HRP (NA934V, GE-Healthcare UK Limited) at 1/1000, and anti-goat IgG-HRP (sc-2020, Santa Cruz Biotechnologies) also at 1/1000.

To quantify the bands obtained via Western blot analysis, we applied band densitometric analysis via ImageJ software (http://rsb.info.nih.gov/ij/). The area under curve (AUC) of the specific signal was corrected for the AUC of the loading control. The value for the ‘Solv’ condition was set as 1 and other conditions were recalculated correspondingly to allow ratio comparisons. For the Hsp70 cell lysate ELISA, we used the EKS-700B Hsp70 ELISA kit according to the manufacturer’s instructions (Stressgen).

### LC-MS/MS Analysis

HEK293T cells were transiently transfected with 10µg Flag-tagged GR using the calcium phosphate transfection method. 24 hours after transfection and after induction with solvent or Compound A (10µM) for 3h, cells were lysed either in 1ml NP40 non-denaturing buffer (50 mM Tris.HCl pH 7.5; 125 mM NaCl; 5% glycerol; 0.2% NP40; 1.5 mM MgCl_2_; and cOmplete® protease inhibitor cocktail (1 tablet/50ml buffer)) or 1ml Buffer A (10mM Hepes pH 7.5; 10mM KCl; 1.5mM MgCl_2_; 0.1% NP-40; 0.5mM DTT; and for cOmplete® protease inhibitor cocktail cocktail (1 tablet/50ml buffer)). Input controls (50µl/sample) of either buffer were kept aside for control western blot analyses. Flag-tagged GR was immunoprecipitated with 40µl Flag beads overnight. Cell lysates in NP40 non-denaturing buffer were processed as follows: after 5 washes with 5mM TEAB (triethylammonium bicarbonate) buffer, elution was performed with 600 µl of 100 µg/ml Flag peptide in 5mM TEAB buffer. Cell lysates in buffer A were processed as follows: after 5 washes with NH_4_HCO_3_ (50mM pH 8), elution was done with 450 µl ammoniumhydroxide (NH_4_OH) (1%).

Reagents and solvents were purchased from Sigma-Aldrich (St. Louis, MO, USA), except acetonitrile (Baker, Center Valley, PA, USA) and formic acid (Biosolve, Valkenswaard, The Netherlands), and used without further purification, unless specified. H_2_O for (HPLC) buffers was produced with a Millipore (Billerica, MA, USA) RIOs-DI water purification system, coupled with the MilliQ Reference A^+^ system equipped with a 22 µm filter and a C18 column to remove organic impurities.

Immunoprecipated samples were dried completely in a rotational vacuum concentrator (RVC 2–33 IR, Martin Christ, Osterode am Harz, Germany). The residue was reconstituted in 500 µl of 50 mM TEAB buffer (pH 8) and the resulting solution was heated at 95°C for 10 min. After cooling on ice for at least 5 min, trypsin (2 µg, Sequencing Grade Modified Trypsin, Porcine, Promega, Leiden, The Netherlands) was added and the samples were overnight incubated at 37°C. Samples were again dried completely in a rotational vacuum concentrator and reconstituted in 20 µl of 2% acetonitrile in 0.1% TFA. Samples were centrifuged for 10 minutes at 15000 g, and the supernatant was transferred to a vial for LC-MS/MS analysis.

The samples were analyzed on a LC-MS/MS system consisting of an Ultimate 3000 RSLC nano (Dionex, Amsterdam, The Netherlands) in-line connected to a LTQ Orbitrap Velos (Thermo Fisher Scientific, Bremen, Germany). The sample was loaded on a trapping column (made in-house, 100 µm internal diameter (I.D.)×20 mm, 5 µm beads C18 Reprosil-HD, Dr. Maisch). After back-flushing from the trapping column, the sample was loaded on a reverse-phase column (made in-house, 75 µm I.D×150 mm, 5 µm beads C18 Reprosil-HD, Dr. Maisch) with solvent A (0.1% trifluoroacetic acid, 2% acetonitrile), and were separated with a linear gradient from 2% solvent A’ (0.1% formic acid) to 55% solvent B’ (0.1% formic acid and 80% acetonitrile) at a flow rate of 300 nl/min followed by a wash reaching 100% solvent B.

The mass spectrometer was operated in data-dependent mode, automatically switching between MS and MS/MS acquisition for the ten most abundant peaks in a given MS spectrum. Full scan MS spectra were acquired in the Orbitrap at a target value of 1E6 with a resolution of 60,000. The ten most intense ions were then isolated for fragmentation in the linear ion trap, with a dynamic exclusion of 30 s. Peptides were fragmented after filling the ion trap at a target value of 1E4 ion counts. The mass spectrometer’s nanospray source was expanded with an Active Background Ion Reduction Device (Abird, ESI Source Solutions). From the MS/MS data in each LC run, Mascot Generic Files were created using the Mascot Distiller software (version 2.4.3.3, Matrix Science, www.matrixscience.com/Distiller). While generating these peak lists, grouping of spectra was allowed in Distiller with a maximum intermediate retention time of 30 s and a maximum intermediate scan count of 5 was used where possible. Grouping was done with 0.005 m/z precursor tolerance. A peak list was only generated when the MS/MS spectrum contained more than 10 peaks. There was no de-isotoping and the relative signal to noise limit was set at 2. These peak lists were then searched with the Mascot search engine (MatrixScience) using the Mascot Daemon interface (version 2.4.0, Matrix Science). Spectra were searched against the human subsection of the Swiss-Prot database (version 2013_01 of UniProtKB/Swiss-Prot protein database containing 20,232 human sequence entries). Variable modifications were set to methionine oxidation, pyro-glutamate formation of amino terminal glutamine, and acetylation of the N-terminus and lysine side chains. The mass tolerance on precursor ions was set to 10 ppm (with Mascot’s C13 option set to 1), and on fragment ions to 0.5 Da. The peptide charge was set to 1+,2+,3+ and the instrument setting was put on ESI-TRAP. Enzyme was set to trypsin, allowing 1 missed cleavage, and cleavage was also allowed when arginine or lysine are followed by proline. Only peptides that were ranked one and scored above the threshold score, set at 99% confidence, were withheld. All data management was done by ms_lims [34].

### siRNA Transfection

A549 cells were seeded and the following day transiently transfected using the calcium phosphate precipitation technique as described before [35]. As indicated, cells were either transfected with siRNA control, siRNA GR (NR3C1) or a combination of siRNA HSPA1A and HSPA1B (Dharmacon). Specific sequences and order numbers can be found in Table S1. Cells were incubated with the respective transfection mixtures overnight (16h). The following day, medium was replaced with DMEM, supplemented with 10% fetal calf serum, and cells were left to rest for 24h. Subsequent to the appropriate inductions, total RNA was isolated using TRIzol Reagent (Invitrogen, Life Technologies). To control for siRNA efficiencies, we collected control RNA samples and total protein cell lysates (SDS Sample buffer) of control wells. RNA samples and control protein samples were analyzed, as described above.

### Immunofluorescence

A549 cells, seeded on coverslips and incubated in Optimem (Gibco) for 48h, were induced as indicated. The protocol regarding fixation, permeabilization and staining was described [5]. Endogenous HSF1 or NF-κB p65 was visualized using a specific Ab followed by Alexa Fluor 488 goat α-rabbit IgG (Molecular Probes) secondary Ab. The antibodies directed against HSF1 and NF-κB p65 were purchased via Cell Signaling Technology and Santa Cruz, respectively. Cell nuclei were stained with DAPI (0.4µg/ml). Alexa fluor 488 excitation or UV-illumination via an Axiovert 200M immunofluorescence microscope (Zeiss) allowed recording of HSF1 or NF-κB p65, or DAPI signal, respectively, with a Zeiss Axiocam mMR. Images were processed using Axiovision Release 4.6.3-SP1 software.

Subcellular distribution of endogenous NF-κB p65 was analyzed using ImageJ software integrated density analysis of cells in various images of the same sample. A representative image is shown. The distribution of NF-κB p65 in each cell is expressed as the percentage of nuclear NF-κB p65 on a cell total of 100% NF-κB p65 and total results of each experiment are presented as a scatter dot plot.

### Chromatin Immunoprecipitation (ChIP) Assay

A549 cells were starved in 0% DMEM for 48 hours. After treatments, cells were subjected to a ChIP assay against HSF1, as described [36]. ChIP analysis of endogenous HSF1 was performed with α-HSF1 antibody (Cell Signaling Technology). Normal rabbit IgG (Santa Cruz) was used as a negative control in ChIP. The amount of sonicated protein:DNA complexes, present before immunoprecipitation (IP), is indicated by the input controls. Final DNA samples of bound immunoprecipitated and input fraction were purified via a QIAquick PCR purification kit (QIAGEN). Purified DNA samples, enriched with the immunoprecipitated protein, and input control DNA samples were subjected to qPCR in triplicate. Primer sequences are available upon request. Data obtained from immunoprecipitated samples were corrected for the signal of the respective input control and final integrated results were presented as ‘Relative recruitment’.

### Statistical Analyses

Statistical analyses were performed with a one-way analysis of variance (ANOVA) and Tukey’s multiple comparison post test, an unpaired t-test or a non-parametric Mann-Whitney-U test, where appropriate, using the asymptotic significant P-values. Error bars indicate SD.

All materials and methods, as described in above are also valid for the figures S1, S2, S3, S4, S5, S6, S7, S8, S9, and S10 and tables S1 and S2. Additional materials en methods to understand figures S1, S2, S3, S4, S5, S6, S7, S8, S9, and S10 and tables S1 and S2 are added in support.

## Results

### Compound A is a Selective GR Modulator with NF-κB Repressive Capacity

**Figure 1:**
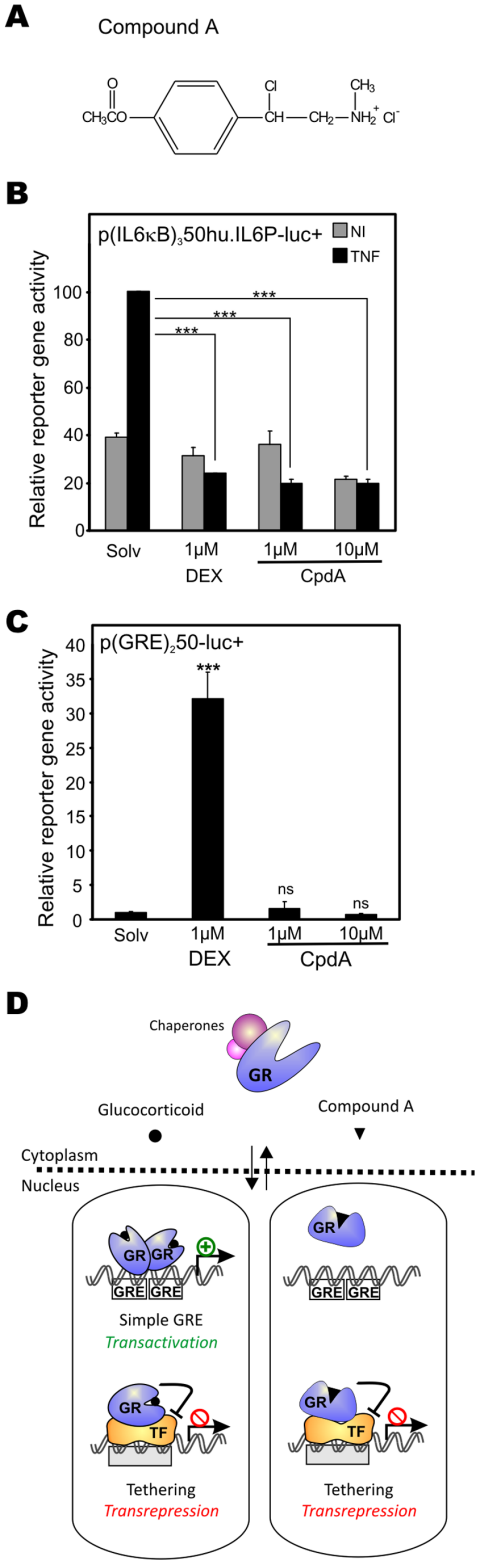
Compound A is a selective GR modulator with NF-κB repressive capacity. (A) Compound A chemical structure (B) A549 cells, transiently transfected with p(IL6κB)_3_50hu.IL6P-luc+, were treated with solvent (Solv), DEX (1µM) or CpdA (1µM or 10µM) for 2h. Subsequently, cells were induced with TNF (2000 IU/ml) for 6h where indicated. β-gal control-corrected results were presented as relative reporter gene activity with the condition Solv/TNF set at 100. Statistical analysis (ANOVA with Tukey’s multiple comparison post test) was performed to show significant difference with the TNF condition for selected conditions (*** p<0.001). (C) A549 cells, transiently transfected with p(GRE)_2_50-luc+, were treated with Solv, DEX (1µM) or CpdA (1µM or 10µM) for 8h. β-gal control-corrected results were presented as relative reporter gene activity with the condition Solv set at 1. Statistical analysis (ANOVA with Tukey’s multiple comparison post test) was performed to show significant difference with the Solv condition (ns not significant; *** p<0.001). Results are shown +/−SD. Figures in (B) and (C) are representative of at least 3 independent experiments. (D) Schematic model of gene modulatory GC and CpdA effects. While classic GCs can transactivate GRE-regulated genes and impede gene expression of specific target genes via a tethering transrepression mechanism (left panel), CpdA drives GR into a monomer formation that does not allow transcactivation of GRE-regulated gene expression. CpdA liganded GR can, however, still repress gene expression via transrepression (right panel).

To confirm the selective GR modulator status of CpdA (or *2-(4-acetoxyphenyl)-2-chloro-N-methyl-ethylammonium chloride* (Figure 1A) in A549 human epithelial cells, we performed reporter gene analyses with transiently transfected cells. The administration of the synthetic glucocorticoid dexamethasone (DEX) or CpdA to a TNF-stimulated NF-κB-driven promoter represses the reporter gene activity in a statistically significant manner (Figure 1B). Alternatively, whereas DEX can potently induce GRE-regulated promoter activity, CpdA has no significant effect on the reporter gene activity of p(GRE)_2_50-luc+ (Figure 1C). Similar data were previously obtained in stably transfected L929sA cells [5]. To sum up, CpdA can be referred to as a selective GR modulator since it represses NF-κB-driven gene expression without inducing the dimeric GR-mediated upregulation of GRE-regulated genes (Figure 1D).

### Both Compound A and Heat Shock Diminish NF-κB-driven Gene Expression and NF-κB Activation

**Figure 2:**
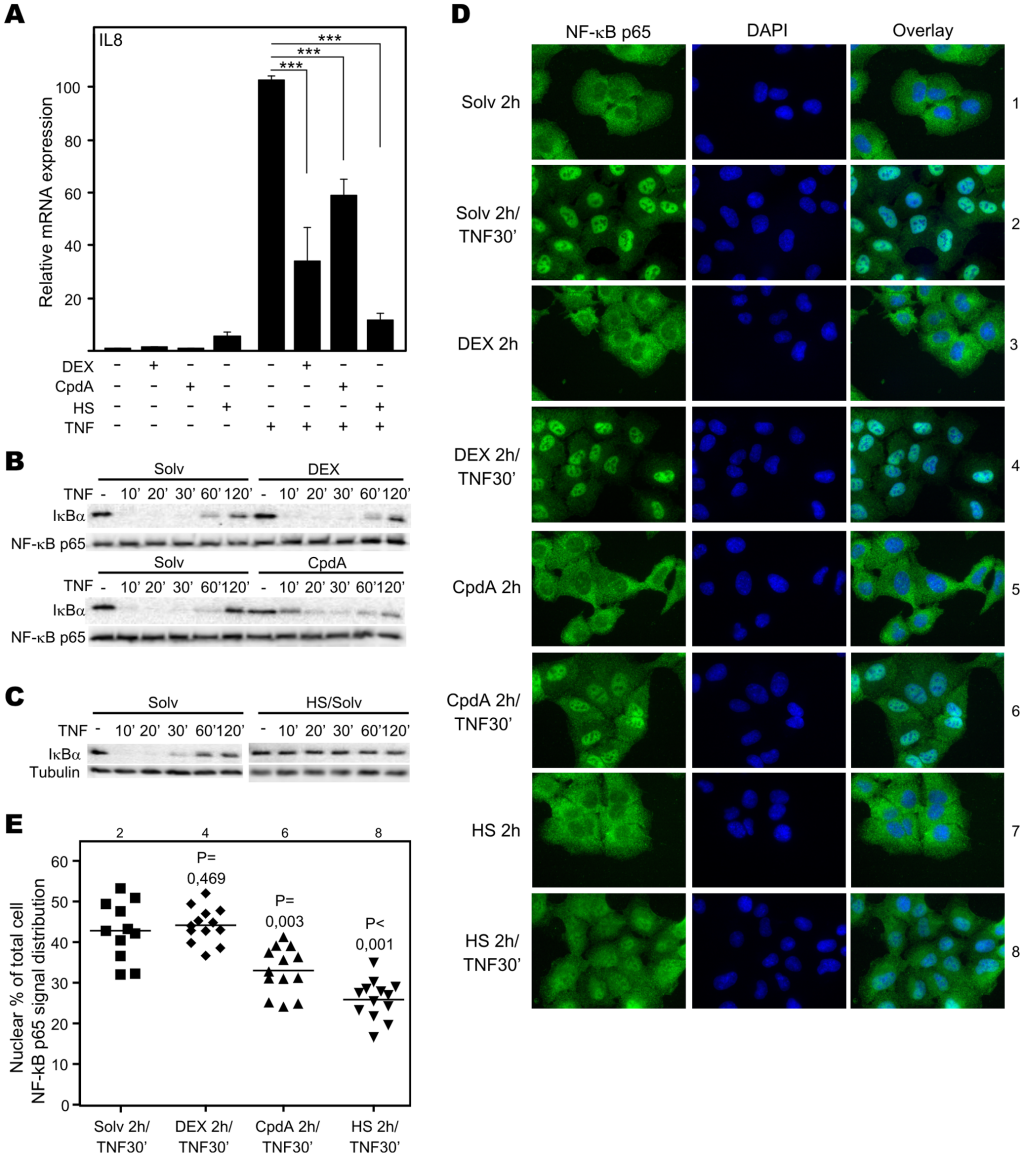
Both Compound A and heat shock diminish NF-κB-driven gene expression and NF-κB activation. (A) A549 cells, starved for 48h, were pretreated for 1.5h with solvent (Solv), DEX (1µM), CpdA (10µM) or subjected to heat shock treatment (1h at 43°C and 30′ recovery at 37°C), ensued with TNF (2000IU/ml) for 5.5h. Isolated total RNA was subjected to RT-qPCR assaying IL8 mRNA levels, normalized to cyclophilin household gene mRNA levels. The TNF condition was set at 100 and results were recalculated accordingly. These results are representative of 2 independent experiments. Statistical analysis (ANOVA and Tukey multiple comparison post test) were performed for selected pair-wise comparisons. (B) A549 cells, starved for 48h, were pretreated for 2h with solvent (Solv), DEX (1µM) or CpdA (10µM), after which TNF(2000IU/ml) was added as indicated. Western blot analysis of total cells lysates detects IκBα protein, with NF-κB p65 as loading control. This figure is representative for 2 independent experiments. (C) A549 cells, starved for 48h, were untreated or heat-shocked at 43°C for 2h. Ensuing, cells were treated by TNF (2000IU/ml) for the indicated times. Total cell lysates were analyzed as in (B), with tubulin as loading control. Results were obtained on 2 separate blots in one experiment. (D) A549 cells, starved for 48h in Optimem, were pretreated for 2h with Solv, DEX (1µM), CpdA (10µM) or 2h heat shock (HS) at 43°C. Subsequently, TNF (2000IU/ml) was added for 30′, where indicated. After washing, fixation, and permeabilization, indirect immunofluorescence detects endogenous NF-κB p65. DAPI staining indicates the nuclei. Additionally, we present overlays. This figure is representative for 2 independent experiments. (E) ImageJ integrated density analysis of the TNF-treated conditions in (D) allows statistical analysis (Mann-Whitney U test) and we show the P-value of comparisons to the Solv/TNF condition. These results are representative of 2 independent experiments.

As it is described that heat shock or elevated Hsp70 can impede NF-κB-mediated transcriptional activity via impairing IκBα proteasomal degradation and limiting the subsequent NF-κB p65 translocation [18], [37], [38], we wondered whether the CpdA-evoked repression of NF-κB-driven gene expression could be mediated by a similar mechanism. Hereto, we first established that all the stimuli we used, i.e. heat shock, CpdA and DEX are able to significantly inhibit TNF-stimulated NF-κB-driven gene expression of IL8 (Figure 2A) and IL6 (Figure S1) in A549 cells. To explore potential mechanistic parallels, we first assayed whether CpdA could impede the TNF-stimulated IKK-mediated IκBα degradation in A549 cells. As expected, TNF addition prompted a complete degradation of IκBα, the level of which starts to rise again after 60′ of TNF treatment (Figure 2B-C). The GC DEX did not affect TNF-induced IκBα degradation in A549 cells (Figure 2B), confirming our previous findings in L929sA cells [32]. In contrast, CpdA administration resulted in an incomplete IκBα degradation after TNF stimulation (Figure 2B). As expected from literature-based evidence in other cell systems, a heat shock treatment, leading to HSF1 activation and highly elevated Hsp70 protein levels, can indeed lead to a total block of the TNF-stimulated proteasomal IκBα degradation (Figure 2C). Because the IκBα-NF-κB p65 association controls the cytoplasmic localization of NF-κB p65 [1], we subsequently investigated the effects of GCs, CpdA and heat shock on the TNF-stimulated NF-κB p65 nuclear translocation in A549 cells. We observed that 30′ of TNF treatment triggers a subcellular shift of NF-κB p65 from the cytoplasm to the nucleus (Figure 2D, lanes 1vs2). Furthermore, we noticed that DEX does not affect the TNF-induced nuclear import of NF-κB p65 (Figure 2D-E: lanes 2vs4). In contrast, pretreatment with CpdA slightly diminishes the TNF-stimulated NF-κB p65 translocation (Figure 2D-E: lanes 2vs6). Furthermore, pre-treatment with heat shock reduces the TNF-induced nuclear translocation of NF-κB p65 in a significant manner (Figure 2D-E: lanes 2vs8). Cytoplasmic localization of NF-κB p65 in unstimulated cells was not altered by induction with DEX, CpdA or heat shock (Figure 2D: lanes 1vs3,5&7). In line with these observations, CpdA hampers the TNF-induced IκBα degradation and/or NF-κB p65 nuclear translocation also in fibroblast-like synovial (FLS) cells [39], primary microglial cells [40] and L929sA fibrosarcoma cells (Figure S2).

To summarize, CpdA reduces the TNF-stimulated IκBα degradation and NF-κB p65 translocation from the cytoplasm to the nucleus. Heat shock treatment totally abolishes TNF-stimulated IκBα degradation and reduces the TNF-induced nuclear translocation of NF-κB 65, more profoundly. Correspondingly, both treatments negatively impact NF-κB-driven gene expression.

### Hsp70 is Required to Allow the anti-inflammatory Activity of Compound A

**Figure 3:**
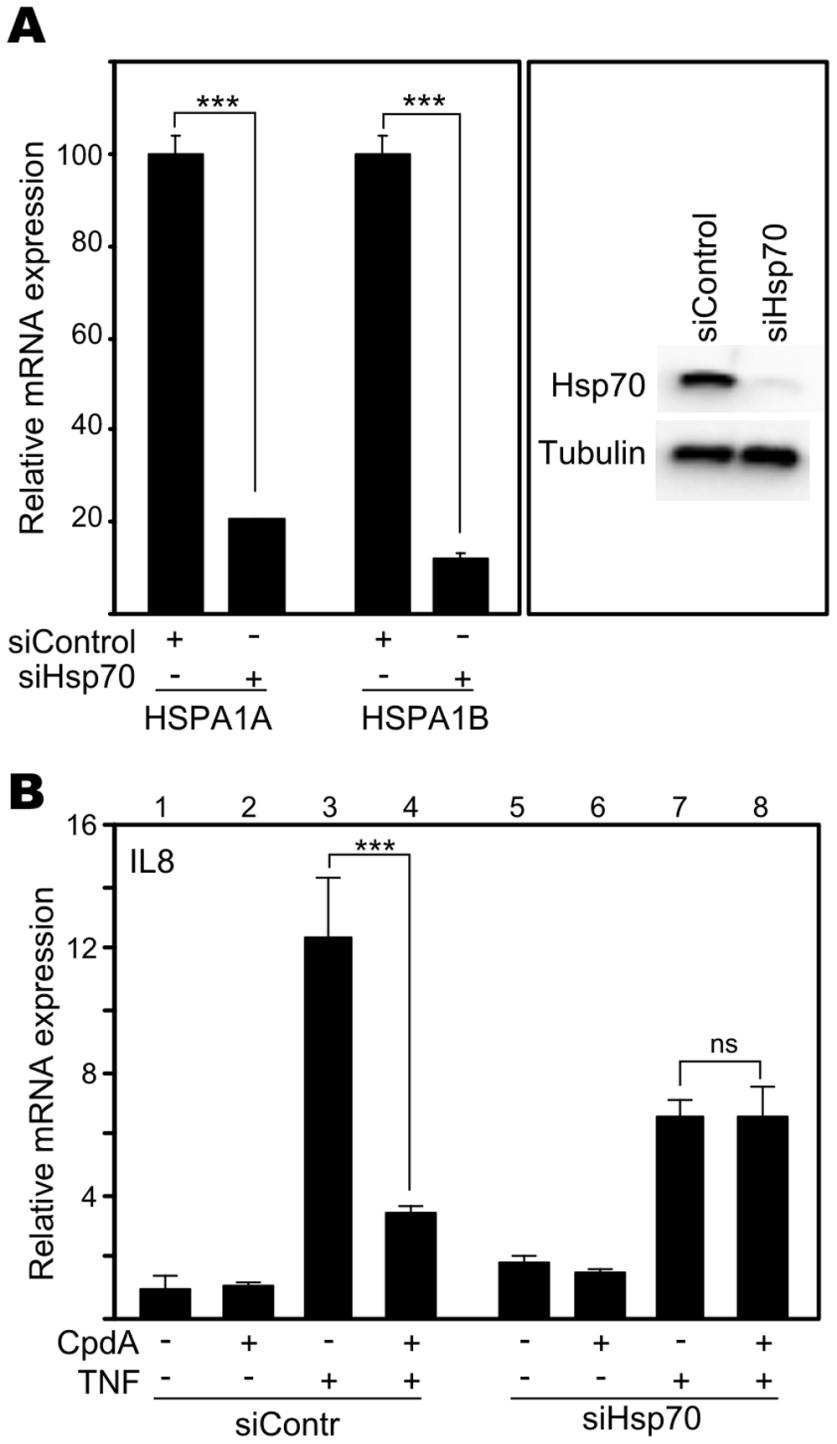
Hsp70 is required to allow the anti-inflammatory activity of Compound A. (A) A549 cells were transfected with siControl or siRNA targeting HSPA1A and HSPA1B (siHsp70). Total RNA or total protein extracts were prepared 48h post transfection. Purified mRNA was subjected to RT-qPCR detecting HSPA1A or HSPA1B gene expression levels and specific results were normalized to housekeeping controls cyclophilin and 28S, as presented in the left panel. For siControl-transfected samples gene expression levels were set at 100%. SiHsp70-transfected results were recalculated accordingly. Statistical analysis (unpaired t-test) was performed to show significant difference between siControl and siHsp70 conditions (*** p<0.001). In the right panel, total cell lysates were subjected to Western blot analysis to detect Hsp70 protein levels. Detection of tubulin served as loading control. (B) In parallel with (A), A549 cells were transfected with siControl or siHsp70. 41h post transfection, cells were pretreated with Solv or CpdA (10µM) for 2h, after which ensued a 6h TNF (2000IU/ml) treatment. Purified mRNA was subjected to RT-qPCR detecting IL8 gene expression levels and specific results were normalized to housekeeping controls cyclophilin and 28S. The condition Solv (siControl) was set as 1 to allow ratio comparisons. Statistical analysis (ANOVA with Tukey’s multiple comparison post test) was performed to show significant difference for selected pair wise comparisons (ns not significant; ** p<0.01; *** p<0.001).

To specifically address the possible role of Hsp70 in the CpdA-mediated anti-inflammatory action mechanism, we knocked down Hsp70 in A549 cells via transfection of siRNA HSPA1A and siRNA HSPA1B, targeting Hsp70 mRNA. The distinct genes HSPA1A and HSPA1B, located in close proximity on chromosome 6 and displaying a 98% sequence similarity, both code for Hsp70 protein. Tests to validate the efficacy of specific siRNA targeting Hsp70 in comparison to non-targeting siControl showed via RT-qPCR that HSPA1A and HSPA1B mRNA levels were significantly knocked down (Figure 3A, left panel) and that, consequently, Hsp70 protein was nearly completely abolished (Figure 3A, right panel). Analysis of TNF-stimulated IL8 gene expression levels demonstrated, as expected, that CpdA can significantly inhibit IL8 gene transcription when cells were transfected with non-targeting siControl (Figure 3B: lanes 3vs4). However, when cellular Hsp70 levels were knocked down via transfection of Hsp70-targeting siRNA, CpdA completely failed to exert its anti-inflammatory effect on IL8 gene expression levels (Figure 3B: lanes 7vs8). Similar results were obtained for IL6 gene expression levels (Figure S3). In summary, adequate cellular levels of Hsp70 protein are required to allow CpdA-mediated repression of pro-inflammatory gene expression.

### Compound A Augments Hsp70 Gene Expression

**Figure 4:**
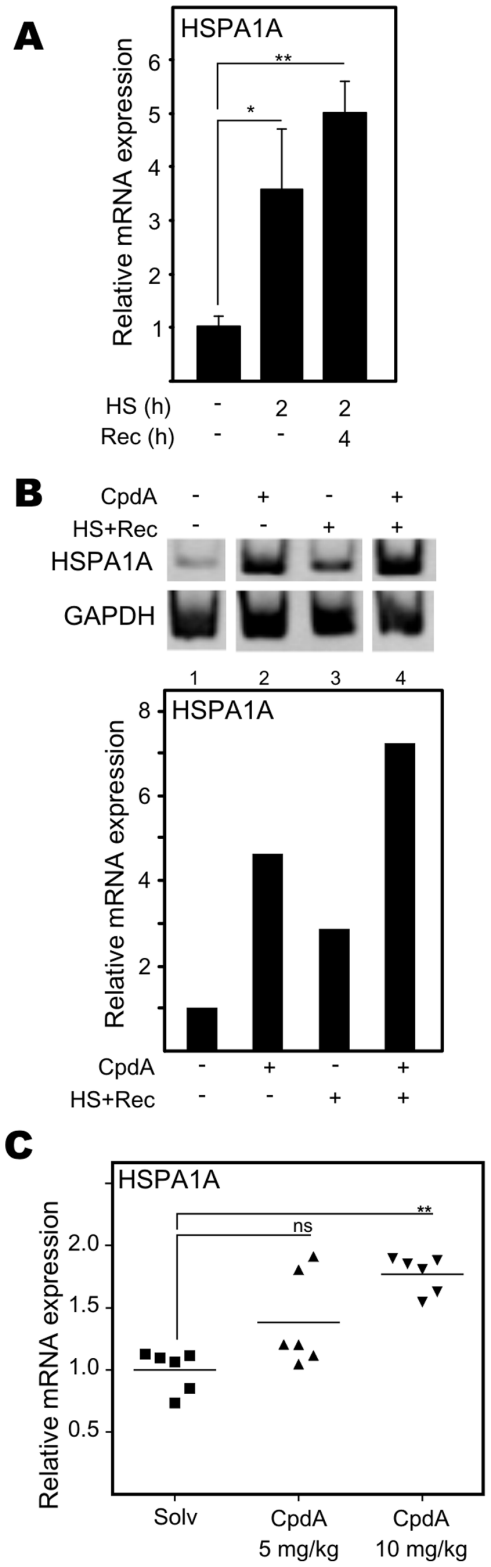
Compound A augments Hsp70 gene expression. (A) A549 cells were left untreated or were induced with heat shock (HS) at 43°C for 2h either or not followed by 4h recovery time (Rec) at 37°C. Isolated total RNA was subjected to RT-qPCR for the detection of HSPA1A, normalized to cyclophilin housekeeping control. The non-induced condition was set as 1 to allow ratio comparisons. Statistical analysis (ANOVA with Tukey’s multiple comparison post test) was performed for selected pair wise comparisons (ns not significant; *p<0.05; ** p<0.01). These results are representative of three independent experiments. (B) A549 cells, treated with solvent (Solv) or CpdA (10µM), were either incubated at 37°C for 6 hours or subjected to the following temperature protocol : 2 hours at 37°C (pre-induction), followed by 2 hours at 43°C heat shock (HS) and lastly 2 hours at 37°C (recovery; Rec). Isolated total RNA was subjected to RT-PCR for the detection of HSPA1A and control GAPDH gene expression levels. The displayed bands were detected from one single gel. The respective bands were quantified using ImageJ software and normalized to GAPDH control expression levels. Solv was set as 1 and all other conditions were recalculated relative to this condition and expressed as relative mRNA expression level. The figure is representative for 2 independent experiments. (C) Eight week old female BALB/c mice were injected intraperitoneally with either PBS as a control or CpdA dissolved in PBS (5mg/kg or 10mg/kg) and 24h later total skin samples were resected and their respective mRNA samples were subjected to RT-qPCR analysis assaying for HSPA1A gene expression levels and normalized to RPL13a, HMBS and ACTB housekeeping controls. The non-induced condition was set as 1 to allow ratio comparisons. Statistical analysis (Mann Whitney-U-test) was performed for selected pair wise comparisons (ns not significant; ** p<0.01).

Since CpdA clearly shows parallels to heat shock with regard to its anti-inflammatory mechanism and since CpdA’s anti-inflammatory mechanism relies on the presence of Hsp70, we studied the effect of CpdA on Hsp70 gene expression. First, we checked whether the selected primers could indeed detect heat shock-inducible Hsp70 and whether the cell system is susceptible to heat shock-induced Hsp70 upregulation. Total RNA was isolated from A549 cells and resulting samples were subjected to RT-PCR for the detection of HSPA1A mRNA levels. Detection of GAPDH mRNA levels served as control for input RNA and RT efficiency. From Figure 4A it is clear that in A549 cells heat shock clearly leads to an increase in HSPA1A gene transcription levels, which increases even more if cells are left to recover at 37°C. Next, we found that CpdA indeed increases the expression of HSPA1A in A549 cells (Figure 4B). Of note, HSPA1A gene expression levels are even higher when the cells were stimulated with CpdA combined with heat shock than when the cells were induced with heat shock alone (Figure 4B), pointing to possible different and hence additive molecular induction mechanisms. Furthermore, CpdA could also elevate the mRNA levels transcribed of the related HSPA2 and HSPA6 genes (Figure S4). Concisely, CpdA can substantially elevate various Hsp70 gene expression levels in A549 cells. Further confirmation of these data was found in the human breast carcinoma cell line MCF-7 (Figure S5) and *in vivo* (Figure 4C). Indeed, complete skins from i.p. injected BALB/c mice were harvested after 24h of induction. As expected, also in these samples, Compound A concentration-dependently enhances HSPA1A mRNA levels (Figure 4C), overall suggesting a cell- and species-independent effect.

### *De novo* Hsp70 production is not Necessary for CpdA’s anti-inflammatory Action Mechanism

**Figure 5:**
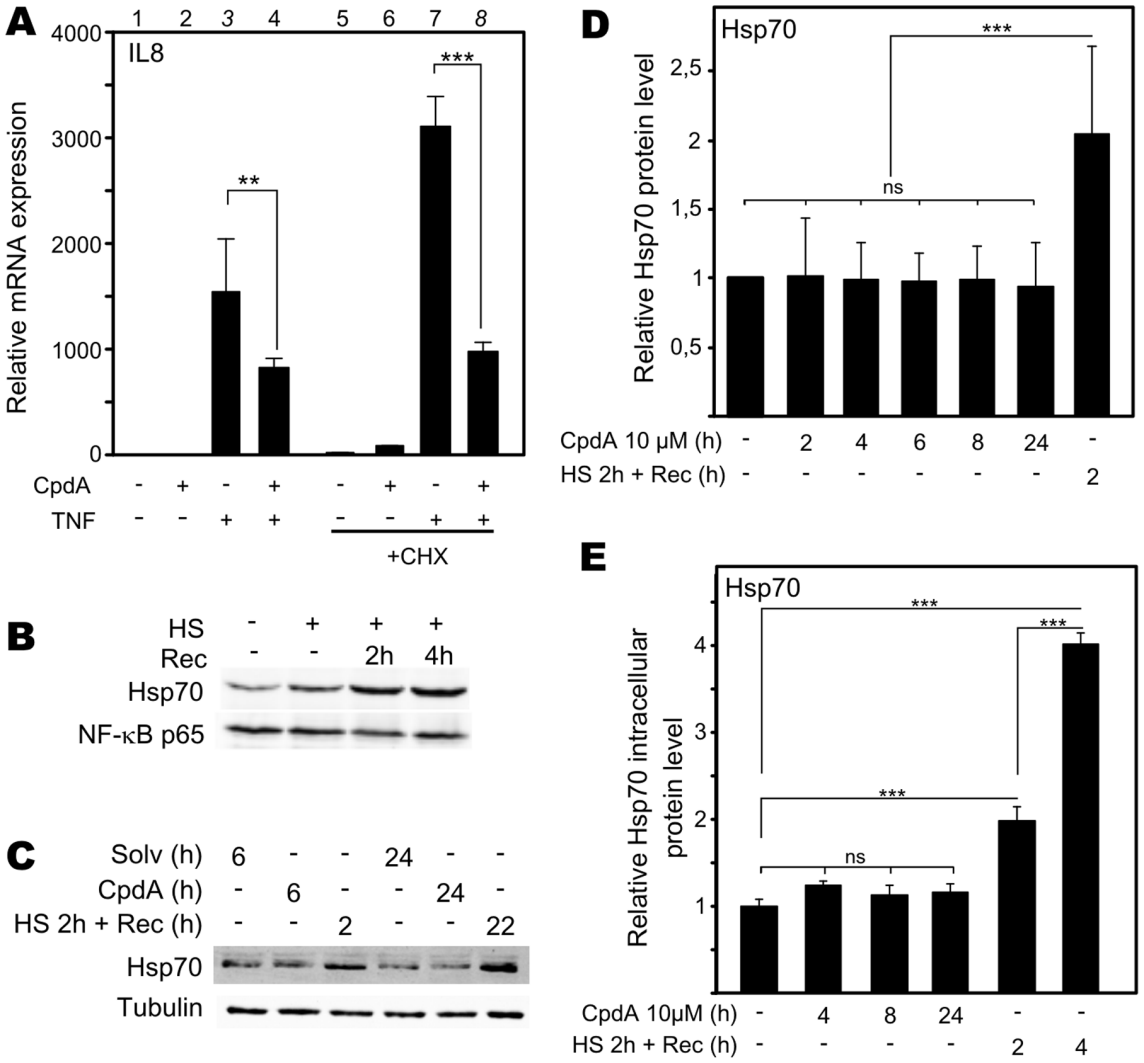
Hsp70 production is not necessary for CpdA’s anti-inflammatory action mechanism. (A) A549 cells, starved for 48h, were left untreated or were pretreated for 30′ with cycloheximide (CHX)(20µg/ml), after which solvent (Solv) or CpdA (10µM) was added for 1.5h. Subsequently, cells were incubated with TNF(2000IU/ml) for 5h. Isolated total mRNA was subjected to RT-qPCR detecting IL8, normalized to GAPDH levels. Solv was set as 1 and other conditions were recalculated accordingly. Statistical analysis (ANOVA with Tukey’s multiple comparison post test) was performed for selected pair wise comparisons (**p<0.001; **p<0.001). This figure is representative for 2 independent experiments. (B) A549 cells were heat-shocked (HS) at 43°C for 2h either or not followed by recovery time (Rec) at 37°C. (C) A549 cells were treated with Solv or CpdA (10µM) for 6h or 24h or heat-shocked at 43°C for 2h, after which cells were left to recover at 37°C for 2h (HS+Rec). (B)(C) Total cell protein extracts were subjected to Western blot analysis detecting Hsp70, with NF-κB p65 or tubulin as loading controls. These images are representative for at least 3 independent experiments. (D) shows the averaged band densitometric analysis (ImageJ) of 8 independent Hsp70 Western blot analyses. Specific Hsp70 signal was corrected for sample loading. Solv was set as 1 to allow ratio comparisons. Statistical analysis (ANOVA with Tukey’s multiple comparison post test) was performed for selected pair wise comparisons (ns not significant; ***p<0.001). (E) A549 cells were treated with solvent or CpdA (10µM) for 4h,8h or 24h or heat-shocked at 43°C for 2h, after which cells were left to recover at 37°C for 2h or 4h(HS+Rec). Total cell protein lysates were analyzed via Hsp70 ELISA. Statistical analysis (ANOVA with Tukey’s multiple comparison post test) was performed for selected pair-wise comparisons (ns not significant; ***p<0.001). This figure represents averaged data of 4 independent experiments.

Since CpdA is able to stimulate Hsp70 gene transcription, we went on to investigate whether CpdA-induced Hsp70 protein synthesis is required for the anti-inflammatory activity of CpdA. Hereto, we treated A549 cells with cycloheximide (CHX), an inhibitor of translational elongation and thus new protein synthesis and then analyzed the anti-inflammatory potential of CpdA toward the TNF-stimulated IL8 gene expression. Remarkably, the CpdA-mediated repression of TNF-induced IL8 gene expression was maintained when new protein synthesis was blocked via CHX (Figure 5A). Functionality of CHX could be confirmed by a Western blot experiment ran in parallel, in which we visualized the short-lived β-catenin protein (Figure S6).

Ensuing, we explored the effects of CpdA on Hsp70 protein levels. First, we confirmed that heat shock treatment elevated Hsp70 protein production in A549 cells (Figure 5B). In correspondence with Figure 4A, the detected Hsp70 Western blot signal was augmented by heat shock treatment and increased further, when cells were left to recover at 37°C (Figure 5B). However, contrary to our expectations, 6h or 24h CpdA treatments of A549 cells did not appear to elevate Hsp70 protein levels (Figure 5C). Densitometric quantification analysis of multiple Western blot analyses clearly confirmed these results (Figure 5D). Moreover, independent Hsp70 ELISAs corroborated this yet again (Figure 5E). Similar data via Hsp70 ELISA were obtained from L929sA cell lysates (Figure S7). Regardless of the fact that Compound A appears unable to elevate the Hsp70 protein level, every assay shows a robust increase in Hsp70 protein levels after heat shock treatment.(Figure 5C-E, Figure S7).

**Figure 6:**
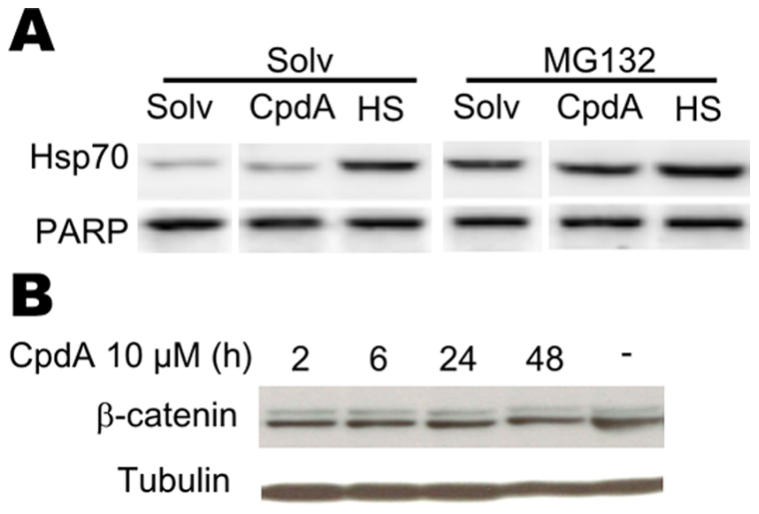
Compound A does not affect proteasomal degradation or translation. (A) A549 cells were preinduced with Solv or MG132 (5µM) for 1.5 h. Subsequently, cells were stimulated with Solv or CpdA (10µM) for 6h or subjected to the following temperature protocol : 2 hours at 43°C followed by 2 hours at 37°C (HS+Rec), as indicated. Total cell protein extracts were subjected to Western blot analysis detecting inducible Hsp70. Detection of PARP served as a loading control. The displayed bands were detected from one single membrane. (B) A549 cells were starved for 48h in 0% DMEM, after which these cells were treated with solvent for 48h or Compound A (CpdA) (10µM) for 2h, 6h, 24h or 48h. Total cell protein extracts were subjected to Western blot analysis detecting β-catenin. Tubulin detection served as a loading control.

To explore whether the lack of a CpdA-induced Hsp70 protein level rise can be attributed to a rapid degradation of the Hsp70 protein, we analyzed A549 and PC-3 cells cells using co-treatments with MG132, an inhibitor of proteasomal degradation. We could show that addition of MG132 does not allow for a CpdA-mediated increase in Hsp70 protein (Figure 6A). To investigate whether CpdA enforces a general block on translation, we analyzed the effects of CpdA on the protein levels of β-catenin, a protein with a short half-life of approximately 2–3h, depending on the cell type [41], [42], [43] (Figure 6B). Treatment of A549 cells with CpdA does not result in a degradation of β-catenin, not even after a 48h treatment with CpdA (Figure 6B). Similar results were obtained in PC-3 cells (Figure S8A). Additionally, we explored whether or not CpdA could affect the expression levels of the constitutively expressed galactosidase reporter gene in stably transfected L929sA cells. As expected, CpdA does not significantly affect these galactosidase levels, again indicating that CpdA does not inhibit translation in general (Figure S8B).

In summary, the anti-inflammatory mechanism of CpdA does not appear to depend on *de novo* (Hsp70) protein synthesis.

### The Glucocorticoid Receptor Interacts with Hsp70 and Hsp90

**Figure 7:**
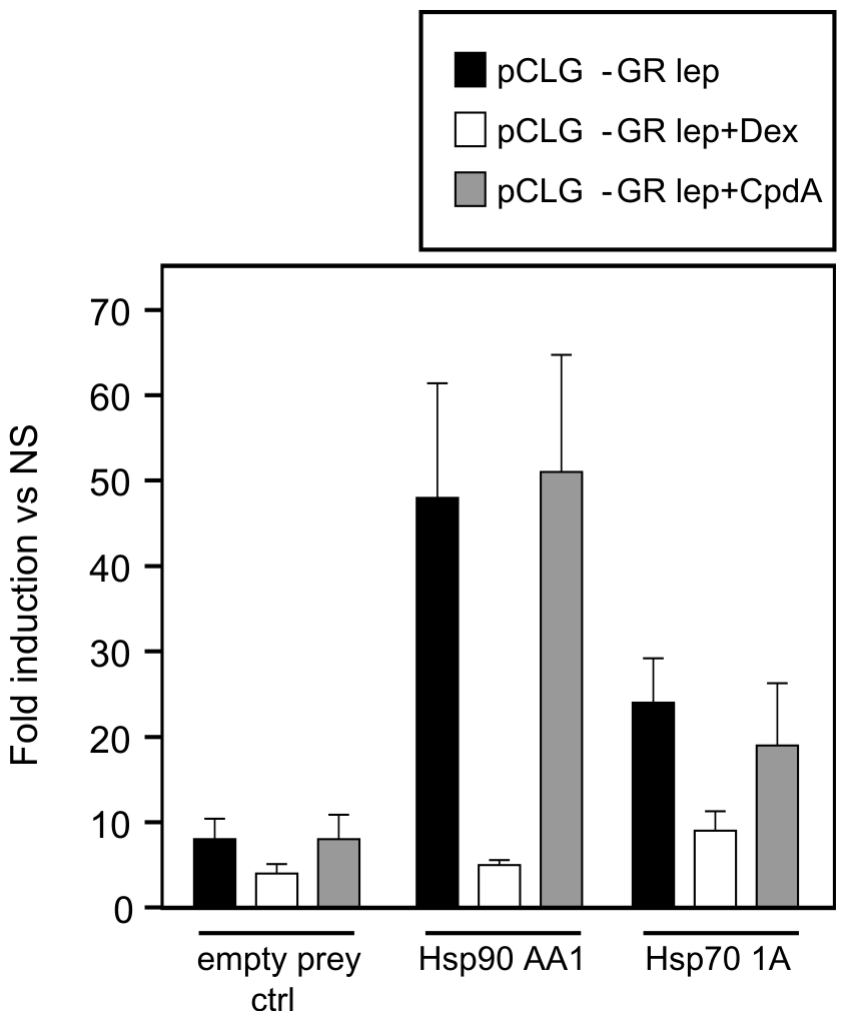
The glucocorticoid receptor interacts with Hsp70 and Hsp90. HEK293T cells were transiently co-transfected with the MAPPIT GRα-bait plasmid, the STAT3-responsive rPAP1-luci reporter plasmid and prey-plasmids as indicated. Empty prey is used as a negative control. Twenty-four hours after transfection cells were stimulated with leptin (100ng/ml) and leptin in combination with DEX (1µM) or CpdA (10µM) for another 24h or were left untreated (NS). Luciferase measurements were performed in triplicate. Data are expressed as mean fold induction (ratio stimulated/untreated).

Mammalian Protein-Protein Interaction Trap (MAPPIT) is a two-hybrid interaction mapping technique based on the functional complementation of a type I cytokine receptor signaling pathway, using a STAT3-dependent luciferase reporter gene as a read-out. MAPPIT relies on a dysfunctional JAK-STAT signaling pathway, of which the activity is only restored when a protein–protein interaction between specific ‘bait’ and ‘prey’ chimeras occurs [44]. Since it operates in intact human cells, MAPPIT allows for analysis of protein:protein interactions under different physiological conditions, e.g. untreated versus DEX- or CpdA-treated cells. As expected, the interaction between unliganded GRα and Hsp90 or Hsp70 (Figure 7) yielded a strong signal in MAPPIT. These interactions were lost upon stimulation with DEX, as expected, but retained upon CpdA induction (Figure 7). There was no interaction (no signal) with the irrelevant bait, confirming that the observed interactions are indeed GRα-specific.

These findings were verified via mass spectrometry analysis, using a shotgun proteomics approach, following the elution of immunoprecipitated Flag-tagged GRα that was overexpressed in HEK293T cells. Table 1 demonstrates that the number of identified MS/MS spectra for associated Hsp70 and Hsp90 proteins was comparable between solvent-treated and CpdA-treated cells (Table 1 and Table S2, listing per identified heat shock protein the corresponding peptides as well as the number of MS/MS spectra per identified peptide). Taken together, CpdA does not lead to dissociation of Hsp70 from the Glucocorticoid Receptor.

**Table 1:** Overview of identified heat shock proteins via LC-MS/MS.

Identified proteins	Number of spectra	Run
Heat shock 70 kDa protein 1A/1B (HSP71_HUMAN)	99	CpdA (FLAG)
Heat shock 70 kDa protein 1A/1B (HSP71_HUMAN)	48	CpdA (NH_4_OH)
Heat shock 70 kDa protein 1A/1B (HSP71_HUMAN)	52	NI (FLAG)
Heat shock 70 kDa protein 1A/1B (HSP71_HUMAN)	50	NI (NH_4_OH)
Heat shock cognate 71 kDa protein (HSP7C_HUMAN)	45	CpdA (FLAG)
Heat shock cognate 71 kDa protein (HSP7C_HUMAN)	45	CpdA (NH_4_OH)
Heat shock cognate 71 kDa protein (HSP7C_HUMAN)	43	NI (FLAG)
Heat shock cognate 71 kDa protein (HSP7C_HUMAN)	37	NI (NH_4_OH)
Heat shock protein HSP 90-alpha (HS90A_HUMAN)	1	CpdA (FLAG)
Heat shock protein HSP 90-alpha (HS90A_HUMAN)	1	CpdA (NH_4_OH)
Heat shock protein HSP 90-beta (HS90B_HUMAN)	8	CpdA (FLAG)
Heat shock protein HSP 90-beta (HS90B_HUMAN)	7	NI (FLAG)
Heat shock protein HSP 90-beta (HS90B_HUMAN)	3	NI (NH_4_OH)

HEK293T cells were transfected with Flag-hGRα via calcium phosphate and following stimulation with either solvent (NI) or 10μM CpdA (CpdA), immunoprecipitated using Flag beads (plasmid and methodology described in [7]. Two identical set-ups were done in parallel, yet, using two different elution methods, via NH_4_OH, as indicated, or via the Flag peptide (FLAG, 100μg/ml). The Swiss-Prot accession is indicated in the protein description field. Per protein, the number of identified MS/MS spectra (at 99% confidence settings (Mascot)) is indicated.

### Compound A Increases Hsp70 Promoter Activity Dose-dependently and Transiently

**Figure 8:**
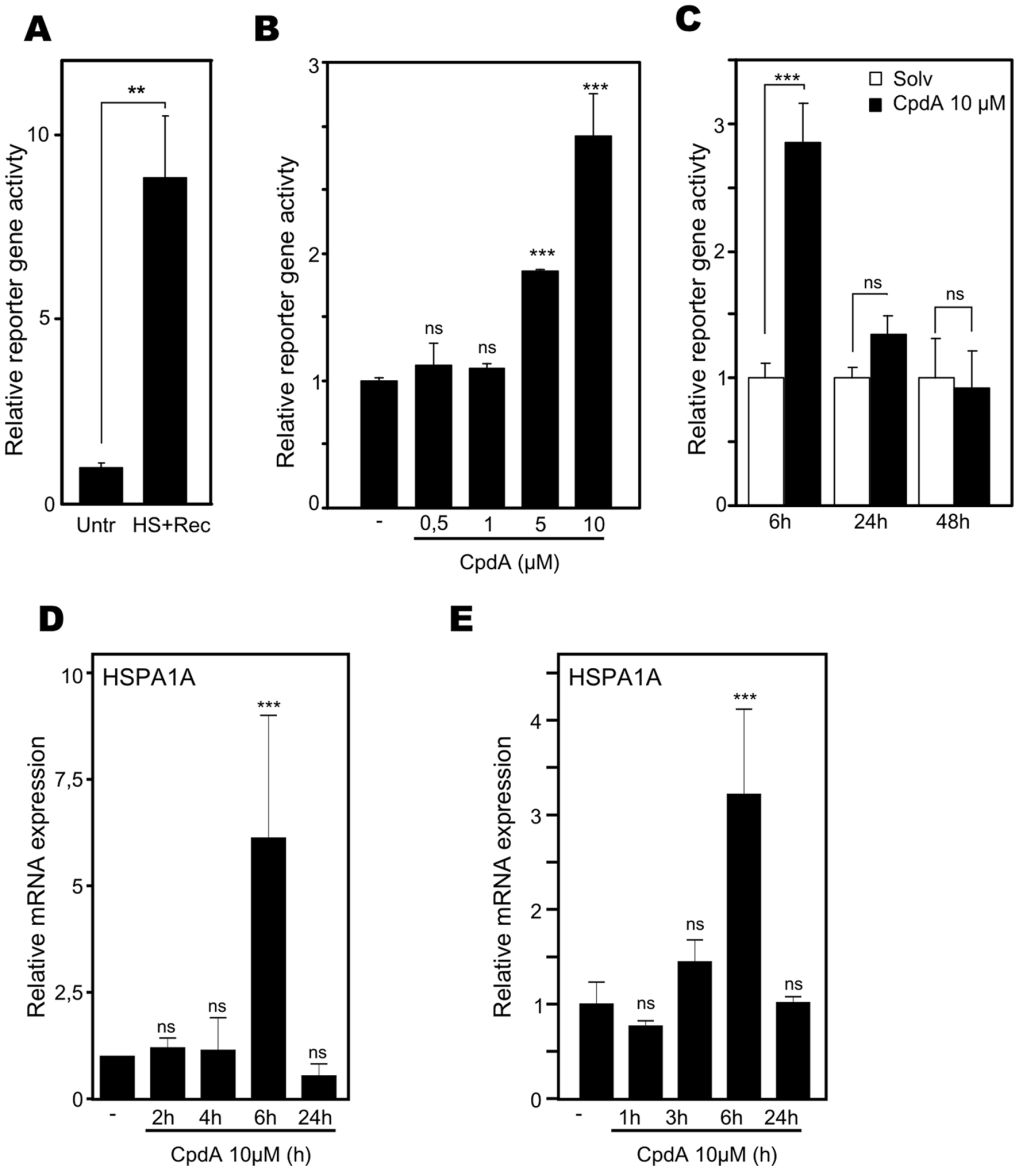
Compound A increases Hsp70 promoter activity dose-dependently and transiently. (A) L929sA cells, stably transfected with a mHsp70i-luc reporter gene construct, were left untreated (Untr) or were stimulated with heat shock at 43°C for 2h, followed by a recovery period of 2h at 37°C (HS+Rec). Normalized luciferase levels were presented as relative reporter gene activity with the condition ‘Untr’ set as 1. Statistical analysis (unpaired t-test) was performed. This figure is representative for 6 independent experiments. (B) L929sA cells, stably transfected with mHsp70i-luc, were induced for 8h with various concentrations of CpdA, as indicated. All samples were controlled to a similar amount of solvent. This figure is representative for 4 independent experiments. (C) L929sA cells, stably transfected with mHsp70i-luc, were treated with solvent (Solv) or CpdA (10µM) for 6h, 24h or 48h. Data were presented as relative reporter gene activity with the Solv condition set as 1. Statistical analysis (ANOVA with Tukey’s multiple comparison post test) was performed to explore if Solv differs from CpdA treatment for each respective induction time. (D) A549 cells, were treated with solvent or CpdA 10µM for the indicated time period. Total cellular mRNA was subjected to RT-qPCR detecting gene expression levels for HSPA1A, normalized using housekeeping 36B4 and β-actin mRNA levels. Four independent experiments with slightly varying time kinetics all show comparable results. (E) PC-3 cells were starved for 48h in 0% DMEM, after which these cells were left untreated or treated with Compound A (10µM), as indicated. Purified mRNA was subjected to RT-qPCR detecting HSPA1A gene expression levels and specific results were normalized to housekeeping controls cyclophilin, GAPDH and 36B4. (B)(D)(E) Solv condition was set as 1 and results recalculated accordingly. Statistical analysis (ANOVA with Tukey’s multiple comparison post test) was performed to compare with Solv (ns not significant; ** p<0.01; *** p<0.001).

Although an increase in Hsp70 protein does not seem to play a role in CpdA’s overall anti-inflammatory mechanism, we were intrigued by the rarely reported gene stimulatory effect of CpdA. Therefore, we decided to explore the promoter regulation of Hsp70 in more detail. To directly investigate the effects of CpdA on Hsp70 promoter activity, we created a L929sA cell line in which the inducible murine Hsp70i-luc reporter gene construct was stably integrated in the genome. First, we tested for heat shock inducibility of the system and could confirm that the stably transfected mHsp70i-luc reporter gene construct responded positively to a heat shock stimulus (Figure 8A). Next, we evaluated the response of this inducible system to CpdA stimulation. We could demonstrate that CpdA concentration-dependently increases Hsp70 promoter activity, showing a significant elevation from a 5µM concentration onwards (Figure 8B). Furthermore, experimenting with variable induction times shows that a 6h CpdA treatment of the L929sA cells, stably transfected with mHsp70i-luc, significantly augments Hsp70 promoter activity, whereas incubations with CpdA for 24h or 48h did not affect Hsp70 promoter activity (Figure 8C). This transient time frame was confirmed when assaying A549 mRNA coding for HSPA1A (Figure 8D) and HSPA2 and HSPA6 (Figure S4), confirming and validating our earlier RT-PCR data (Figure 4B). Similar data were also obtained in PC-3 prostate adenocarcinoma cells (Figure 8E). In summary, Hsp70 promoter activity is concentration-dependently and transiently enhanced by CpdA in different cell lines and species.

### CpdA stimulation of the Hsp70 gene promoter occurs via a GR-dependent mechanism

**Figure 9:**
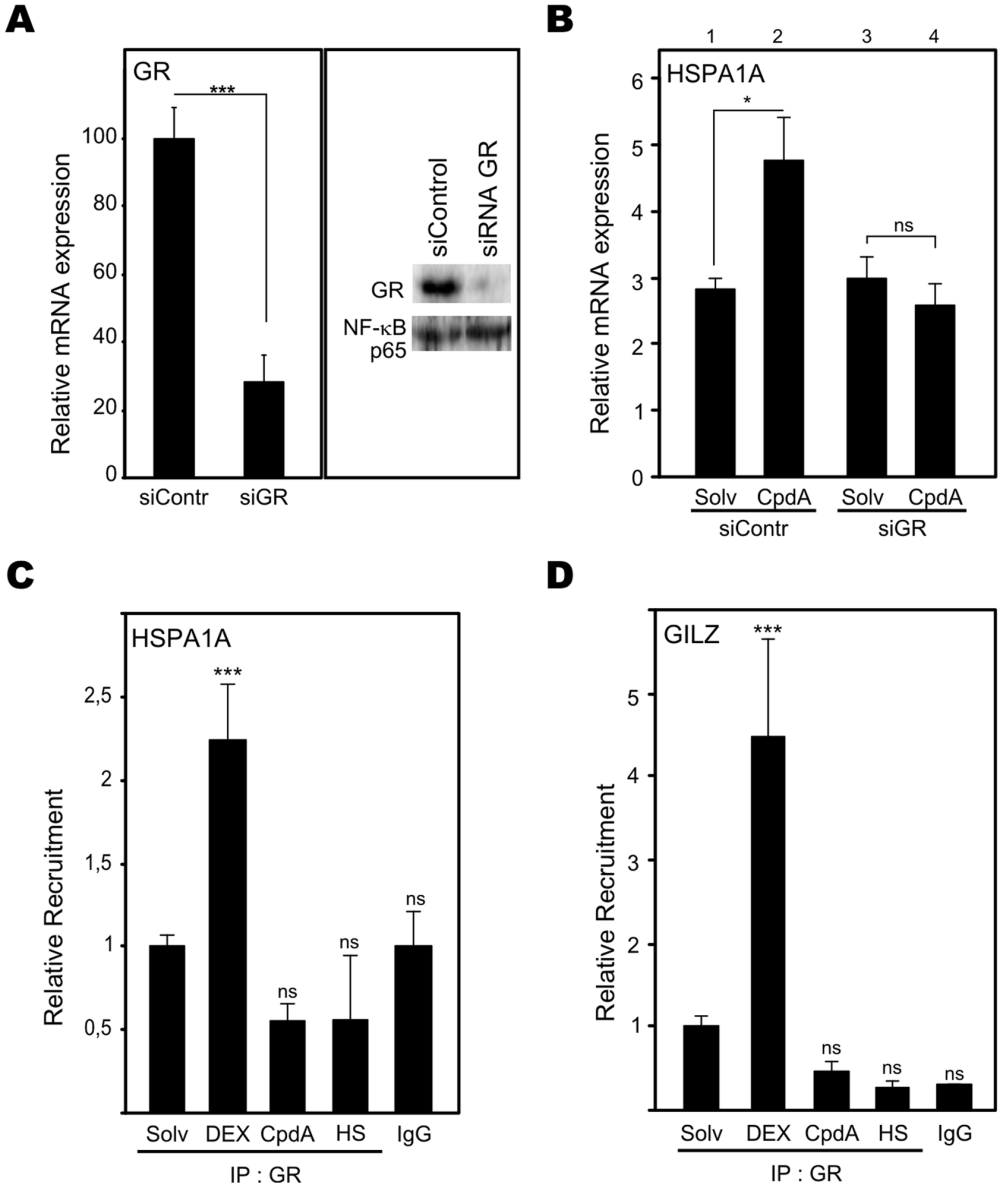
CpdA stimulation of the Hsp70 gene promoter occurs via a GR-dependent mechanism. (A) A549 cells were transfected with siControl or siRNA targeting GR (siGR). Total RNA or total protein extracts were prepared 48h post-transfection. In the left panel, purified mRNA was subjected to RT-qPCR detecting GR gene expression levels, normalized to housekeeping controls cyclophilin and 28S. For the siControl-transfected sample, signal was set at 100%. Data from SiGR-transfected cells were recalculated accordingly. Statistical analysis (unpaired t-test) was performed to show significant difference between siControl and siGR conditions (*** p<0.001). In the right panel, total cell lysates were subjected to Western blot analysis to detect GR protein, with NF-κB p65 as a loading control. (B) In parallel with (A) A549 cells were transfected with siControl or siGR. 41h post transfection, cells were induced with Solv or CpdA (10µM) for 8h. The derived purified mRNA was subjected to RT-qPCR detecting HSPA1A gene expression levels and specific results were normalized to housekeeping controls cyclophilin and 28S. The condition Solv (siControl) was set as 1 to allow ratio comparisons. Statistical analysis (ANOVA with Tukey’s multiple comparison post test) was performed for selected pair wise comparisons (ns not significant; * p<0.05). This experiment is representative for 2 independent experiments. (C) and (D) A549 cells, serum-starved for48h in 0% DMEM, were treated with Solv, Dex (1µM), CpdA (10 µM) for 2h, or exposed to a 43°C heat shock (HS) for 1h. Total cell extracts were subjected to a ChIP assay targeting GR. Ensuing, qPCR signal of immunoprecipitated HSPA1A and GILZ gene promoter fragments is presented relative to input data. Binding to rabbit IgG represents aspecific binding. Statistical analysis (ANOVA with Tukey’s multiple comparison post test) was performed to show significant difference with the Solv condition (ns not significant; *** p<0.001). This experiment is representative for 2 independent experiments.

As CpdA is a selective GR modulator, driving GR into a monomeric formation [7], we wondered whether the stimulatory effect of CpdA on Hsp70 gene expression directly relies on the presence of GR. Hereto, we transfected A549 cells with siRNA targeting GR mRNA, resulting in a strong knockdown of GR mRNA and protein levels (Figure 9A). Analysis of HSPA1A gene expression levels in the non-targeting siControl transfected samples showed that induction with CpdA again is able to stimulate HSPA1A gene expression (Figure 9B), once more confirming and validating our RT-PCR data (Figure 4B). However, when we knocked down GR mRNA and protein levels, induction with CpdA failed to stimulate HSPA1A gene expression levels (Figure 9B). Based on these data, CpdA stimulation of HSPA1A gene expression can be considered as a GR-dependent phenomenon. Further analysis was performed at the promoter level via a GR-targeting Chromatin immunoprecipitation (ChIP), looking at promoter sites flanking the HSE locus in the HSPA1A gene promoter and the GRE locus in the glucocorticoid-induced leucine zipper (GILZ) gene promoter. Remarkably, the data revealed that CpdA did not direct the GR to the HSPA1A gene promoter, in contrast to DEX (Figure 9C). Focusing on a classical GRE-regulated GILZ promoter, a similar DEX-, but not CpdA-induced increase in GR occupancy was observed, as expected, confirming the functionality of our assay (Figure 9D). Also heat shock treatment failed to support GR occupancy at the HSPA1A or GILZ gene promoters (Figure 9C-D). In conclusion, CpdA stimulation of the Hsp70 gene promoter occurs via a GR-dependent mechanism, but without GR occupancy at Hsp70-coding gene promoters.

### Compound A Does not Activate HSF1

**Figure 10:**
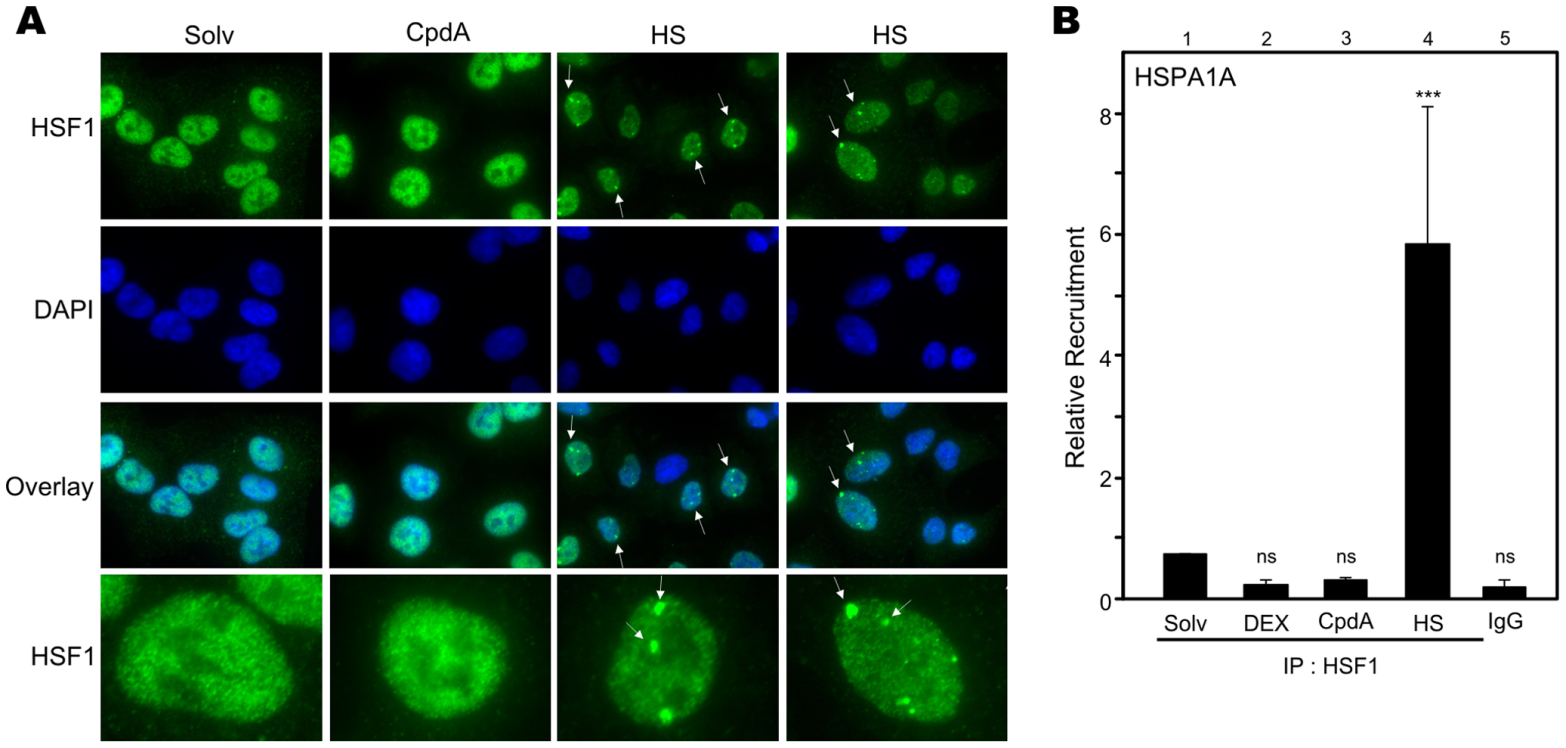
Compound A does not activate HSF1. (A) A549 cells, starved for 48h in Optimem, were treated with solvent (Solv) or CpdA (10µM) for 4h or heat-shocked (HS) at 43°C for 1h. Via indirect immunofluorescence using an α-HSF1 Ab, endogenous HSF1 was visualized (green) and DAPI staining indicates the nuclei of the cells (blue). We also present an overlay and in the panel below, we digitally zoom in on one cell. White arrow heads indicate nuclear stress granules or foci of HSF1. This experiment is representative for 2 independent experiments. (B) A549 cells, starved for 48h in 0% DMEM, were treated with Solv, CpdA (10µM) or DEX (1µM) for 2h or HS at 43°C for 1h. Total cell extracts were subjected to a ChIP assay targeting HSF1. Ensuing, qPCR signal of immunoprecipitated HSPA1A gene promoter fragments is presented relative to input data. Binding to rabbit IgG represents aspecific binding. Statistical analysis (ANOVA with Tukey’s multiple comparison post test) was performed to show significant difference with the Solv condition (ns not significant; *** p<0.001). This experiment is representative for 2 independent experiments.

As heat shock is known to stimulate Hsp70 gene expression via the activation of the transcription factor HSF1, and since GR seems to be an indirect contributor for CpdA-activated Hsp70 gene expression, we hypothesized CpdA may be able to exert effects on HSF1. Therefore, we analyzed via indirect immunofluorescence the endogenous HSF1 protein signal in A549 cells. As expected, we could show that heat shock treatment for 30min (Figure S9) and 1h induces the appearance of bright nuclear foci of HSF1, also called nuclear stress granules, indicated by white arrows (Figure 10A), indicative of an activated HSF1 [12], [45]. In contrast, CpdA treatment for 30min, 1h (Figure S9) and up to 4h did not result in the aggregation of HSF1 in nuclear stress granules (Figure 10A). From Western blot analyses it also became clear that CpdA does not affect HSF1 cellular protein levels (Figure S10A-B). Moreover, CpdA does not shift the HSF1 band to a higher molecular weight, whereas heat shock treatment can induce a shift of HSF1, suggesting intense phosphorylation and thus activation (Figure S10A) [13], [45]. Moreover, when investigating HSF1 recruitment to the proximal HSPA1A gene promoter via ChIP analysis, we could demonstrate that heat shock induces a significant increase in HSF1 recruitment at this site (Figure 10B). In contrast, CpdA did not significantly change HSF1 occupancy at the HSPA1A promoter (Figure 10B). As expected, the GC DEX did not affect basal recruitment of HSF1 at the HSPA1A promoter. The specificity of the HSF1 signal was verified via analysis of binding to rabbit IgG. Taken together, CpdA does not stimulate HSF1 transcription factor activity, in contrast to heat shock treatment.

## Discussion

The selective GR modulator CpdA (Figure 1A) is capable of efficaciously inhibiting NF-κB-driven gene expression while leaving GRE-regulated promoter activity unaffected (Figure 1) [5]. CpdA competes with DEX for GR binding, forcing GR into a monomer formation and evoking GR translocation into the nucleus in various cell lines [8]. The CpdA-mediated mechanism repressing NF-κB-driven gene expression depends on the presence of GR, since CpdA’s anti-inflammatory effect could not be observed in HEK293T cells devoid of a significant amount of endogenous GR [5], and was lost in FLS cells in which GR had been efficiently knocked down [39].

### Role of Hsp70 in CpdA’s anti-inflammatory Mechanism

Hsp70 and activated HSF1 are anti-inflammatory mediators targeting NF-κB in various cell systems [17], [18], [37], [46]. Mechanistically, elevation of the Hsp70 levels targets IKKγ, resulting in a disruption of the IKK complex [18], [37]. Although elevated Hsp70 levels can inhibit, but not abolish, IκBα phosphorylation and does not affect IκBα ubiquitination [18], [38], heat shock induction leading to increased Hsp70 levels impairs IκBα proteasomal degradation and limits the subsequent NF-κB p65 translocation [18], [37], [38]. Additionally, heat shock can elevate IκBα promoter activity and mRNA stability [47], [48]. Furthermore, elevated Hsp70 levels can negatively affect the release of the pro-inflammatory high mobility group box 1 (HMGB1) [38]. Given the mechanistic resemblance between heat shock and CpdA, with respect to their effects on cytokine gene expression, IκBα degradation and NF-κB p65 nuclear translocation (Figure 2, Figure S2) in various cell systems [39], [40], [49], [50], we were not surprised to detect that CpdA can elicit a concentration-dependent, yet transient, rise in Hsp70-coding mRNA levels and promoter activity (Figure 4, 8, Figure S4) in human A549 and PC-3 cells and murine L929sA cells. These findings could be confirmed in human MCF7 breast cancer cells (Figure S5) and skins of BALB/c mice (Figure 4C), indicating species-, cell- and tissue independence of this phenomenon. Knocking down Hsp70 abrogates the ability of CpdA to repress TNF-stimulated IL8 and IL6 gene expression (Figure 3, Figure S3). However, in spite of the clear effect of CpdA on Hsp70 promoter activity and gene expression, no CpdA-instigated elevation in Hsp70 protein was detected (Figure 5B-D, Figure S7). Experiments with the translational elongation inhibitor CHX indicate that CpdA’s anti-inflammatory mechanism does not require *de novo* protein synthesis (Figure 5A) and a persistent association was observed between pre-existing Hsp70 and CpdA-activated GR (Figure 7, Table 1, Table S2). Taken together, these results most likely point to a role of Hsp70 in the GR chaperone complex to mediate the ‘reception’ of CpdA by the GR-Hsp70 complex (Figure 7, Table 1, Table S2), additionally supporting the concept that the CpdA-regulated anti-inflammatory mechanism acts via GR binding and subsequent modulation of GR-dependent phenomena.

The underlying reason for the disconcordance between Hsp70 gene expression and Hsp70 protein production following CpdA treatment is currently unknown. Although secreted Hsp70 has immunomodulatory effects [51], [52], [53] we were unable to find support for a CpdA- or heat shock-induced secreted Hsp70. Furthermore, we investigated whether a rapid proteasomal degradation of Hsp70 [54], [55] could lie at the basis of the difference between protein versus mRNA and reporter gene activity results. As expected, MG132-mediated blocking of proteasomal degradation could elevate basal Hsp70 protein levels substantially, either via the inhibition of Hsp70 protein degradation [54], [55] or also via a HSF1-based stimulation of Hsp70 gene transcription [56], [57], [58], [59]. However, the induction of Hsp70 by CpdA treatment in the presence of MG132 is not enhanced in comparison to the natural CpdA-stimulated fold induction of Hsp70 protein in the absence of MG132 (Figure 6A), indicating that the minor fold increase is not caused by a rapid turn-over of CpdA-induced Hsp70 protein. Furthermore, we could show that CpdA does not enforce a general block on translation as the translation of luciferase (Figure 8B-C) and galactosidase (Figure S8B) in various reporter gene assays is not blocked. Moreover, the protein level of β-catenin, a protein with a short half-life of approximately 2–3h [41], [42], [43] shows no decline in response to CpdA, not even after 48h, in both A549 and PC-3 cells (Figure 6B, Figure S8A). However, the puzzling matter of a CpdA-induced rise in HSPA1A mRNA without the expected rise in Hsp70 protein remains. More than one hypothesis can account for this observation: for example, a sequence-specific inhibition of translation may occur or, alternatively, CpdA might elevate Hsp70 targeting miRNAs [60]. These possibilities would require extensive additional research. The consistent observation that CpdA can induce Hsp70 mRNA gene expression, without elevating Hsp70 protein levels makes us wonder what the precise role of this CpdA-stimulated Hsp70 mRNA might be. It is tempting to speculate that Hsp70 mRNA could serve as a cofactor, conform reports on the steroid receptor RNA activator SRA [61], [62], [63], which acts as a steroid receptor cofactor. Similar to Hsp70, some isoforms of SRA do code for a functional protein, i.e. SRAP. Finally and less exciting, the CpdA-induced production of Hsp70 mRNA may be an unintentional by-product of CpdA-modulated mechanisms. Albeit the question remains why the cell would spend energy on transcribing a mRNA with no further functional implication. Additional research could shed more light on this matter.

### CpdA-mediated Hsp70 Gene Transcription

Stimulation with CpdA combined with heat shock results in higher HSPA1A gene expression levels, than in solely heat shock- or CpdA-induced cells (Figure 4B). This additional Hsp70 mRNA increase could therefore suggest alternative Hsp70 promoter activation mechanisms. The latter hypothesis, potentially implying that heat shock-inducible HSF1 does not act as a mediator of CpdA-mediated Hsp70 upregulation, is clearly supported by the following arguments. First, in contrast to heat shock treatment, CpdA does not enhance HSF1 recruitment to the HSPA1A promoter (Figure 10B). Second, CpdA does not trigger the formation of nuclear stress granules of HSF1, which are indicative for heat shock-induced and activated HSF1 [12], [45] (Figure 10A). In addition, a second mechanistic aspect of the CpdA-mediated Hsp70 increase, was revealed upon investigating GR dependence. Our results strongly suggest that the presence of GR is an absolute requirement for the CpdA-instigated HSPA1A mRNA upregulation (Figure 9B). However, the involvement of GR does not coincide with a recruitment of GR to the HSPA1A gene promoter, suggesting that GR might act as a mediator rather than an effector. Interestingly, it has been described that GCs can repress heat shock-induced Hsp70 gene expression [64], [65], via the interference of GC-activated GR with the recruitment of the transcription factor HSF1 to the proximal HSE-elements in the Hsp70 promoter [65]. These reports provide the context for the observed DEX-mediated recruitment of GR to the HSPA1A gene promoter (Figure 9C). Although stimulation of Hsp70 promoter activity by CpdA is GR-dependent, this mechanism is most likely not a classical GR dimer-driven, GRE-regulated transactivation as CpdA does not mediate GRE-regulated transcription, does not instigate GR dimerization and actively drives GR into a monomer formation [5], [7].

To sum up, CpdA and heat shock use different signalling pathways to induce Hsp70 gene expression. Whereas heat shock activates HSF1, which acts as a transcription factor initiating Hsp70 gene expression, CpdA does not induce HSF1 activation. Additionally, CpdA necessitates the GR to be able to trigger Hsp70 gene expression.

In conclusion, in resemblance to the established anti-inflammatory effect of Hsp70 via halting TNF-stimulated IκBα degradation and NF-κB p65 translocation, we could show that CpdA partially hampers TNF-stimulated IκBα degradation and NF-κB p65 translocation. Correspondingly, CpdA enhances Hsp70 gene promoter activities and transcription, yet without producing additional Hsp70 protein. CpdA’s anti-inflammatory mechanism does not require new protein synthesis and thus new Hsp70 protein production. Nevertheless, the cellular presence of Hsp70 mRNA and protein, most likely as the GR-interacting chaperone, remains crucial for CpdA’s ability to repress NF-κB-driven gene expression. Mechanistically, the selective GR modulator CpdA enhances Hsp70 promoter activity via a HSF1-independent and GR-dependent mechanism, whereas heat shock induces a rise in Hsp70 production via a HSF1-dependent and GR-independent mechanism. These data further support the hypothesis that CpdA is a dissociative modulator of GR, utilizing GR to repress pro-inflammatory promoter activation.

## Acknowledgments

The authors would like to thank I Vanherpe, D. Bracke, B. Gilbert and J Thommis for their excellent technical assistance. Additionally, we thank C. Libert for critical reading of the original manuscript.

## Supporting Information

Figure S1 **Both Compound A and heat shock diminish IL6 gene expression.** (A) A549 cells, starved for 48h, were pretreated for 1.5h with solvent (Solv), DEX (1µM), CpdA (10µM) or subjected to heat shock treatment (1h at 43°C and 30′ recovery at 37°C), ensued with TNF (2000IU/ml) for 5.5h. Isolated total RNA was subjected to RT-qPCR assaying IL6 mRNA levels, normalized to cyclophilin household gene mRNA levels. The TNF condition was set at 100 and results were recalculated accordingly. These results are representative of 2 independent experiments. Statistical analysis (ANOVA and Tukey multiple comparison post test) were performed for selected pair-wise comparisons.
(TIF)

Figure S2 **Compound A diminishes IκBα degradation and NF-κB translocation.** (A) L929sA cells, starved for 48h, were pretreated for 2h with solvent (Solv), DEX (1µM) or CpdA (10µM), after which TNF (2000IU/ml) was added as indicated. Western blot analysis of total cells lysates detects IκBα protein, with NF-κB p65 as loading control. This figure is representative for 2 independent experiments. (B) L929sA cells, starved for 48h in Optimem, were pretreated for 2h with Solvent or CpdA (10µM). Subsequently, TNF (2000IU/ml) was added for 30′, where indicated. After washing, fixation, and permeabilization, indirect immunofluorescence detects endogenous NF-κB p65. DAPI staining indicates the nuclei. Additionally, we present overlays.
(TIF)

Figure S3 **Hsp70 is required to allow the anti-inflammatory activity of Compound A.** A549 cells were transfected with siControl or siRNA targeting HSPA1A and HSPA1B (siHsp70). 41h post transfection, cells were pretreated with Solv or CpdA (10µM) for 2h, after which ensued a 6h TNF (2000IU/ml) treatment. Total RNA extracts were prepared. Purified mRNA was subjected to RT-qPCR detecting IL6 gene expression levels and specific results were normalized to housekeeping controls cyclophilin and 28S. The condition Solv (siControl) was set as 1 to allow ratio comparisons. Statistical analysis (ANOVA with Tukey’s multiple comparison post test) was performed to show significant difference for selected pair wise comparisons (ns not significant; ** p<0.01).
(TIF)

Figure S4 **Compound A augments Hsp70 gene expression.** A549 cells, were treated with solvent or CpdA 10µM for the indicated time period. Total cellular mRNA was subjected to RT-qPCR detecting gene expression levels for HSPA2 or HSPA6 (as indicated), normalized using housekeeping 36B4 and β-actin mRNA levels. The Solv condition was set as 1 and results recalculated accordingly. Statistical analysis (ANOVA with Tukey’s multiple comparison post test) was performed to compare with Solv (ns not significant; ** p<0.01). Three independent experiments with slightly varying time kinetics all show comparable results. (B) MCF-7 breast cancer cells
(TIF)

Figure S5 **CpdA can elevate Hsp70 gene expression levels in MCF7 cells.** (A) MCF7 cells were pretreated with solvent or CpdA (10µM) for 8 h. Total RNA was reverse transcribed and HSPA1A and housekeeping GAPDH mRNA levels were determined via semi-quantitative PCR visualized on a 2% agarose gels. The displayed bands were detected from one single gel. (B) MCF7 cells were assayed via the 'GEarray Q series Analysis with Human Stress and Toxicity pathway' (SABiosciences). Cells were treated with solvent or CpdA (10 µM) for 8h. Total RNA was isolated and reverse transcribed to hybridize labeled cDNA to Human Stress and Toxicity GEA array membranes. Results, visualized via Phospho-Imager, were quantified and controlled for by housekeeping genes. Effects of CpdA are presented as 'fold induction'.
(TIF)

Figure S6 **Control of CHX functionality.** A549 cells, starved for 48h, were left untreated or were treated for 7h with cycloheximide (CHX) (20µg/ml). Total cell protein extracts were subjected to Western blot analysis detecting β-catenin and an aspecific band serves as a loading control.
(TIF)

Figure S7 **CpdA does not elevate the Hsp70 protein level in L929sA cells.** L929sA cells were treated with solvent or CpdA (10µM) for 4h,8h or 24h or heat-shocked at 43°C for 2h, after which cells were left to recover at 37°C for 2h (HS+Rec). Total cell protein lysates were analyzed via Hsp70 ELISA. Statistical analysis (ANOVA with Tukey’s multiple comparison post test) was performed for selected pair-wise comparisons (ns not significant; **p<0.01). This figure represents averaged data of 2 independent experiments.
(TIF)

Figure S8 **Compound A does not block translation.** (A) PC-3 cells were starved for 48h in 0% DMEM, after which these cells were treated with solvent for 48h or Compound A (CpdA) (10µM) for 2h, 6h, 24h or 48h. Total cell protein extracts were subjected to Western blot analysis detecting β-catenin. Tubulin detection served as a loading control. (B) L929sA cells, stably transfected with p(IL6κB)_3_50hu.IL6P-luc+, were left untreated (NI), or were treated with solvent (Solv), or CpdA (0.1µM, 1µM or 10µM) for 8h. The relative activity of the constitutively expressed galactosidase (β-gal) controls were presented as relative reporter gene activity with the condition Solv set at 100. All other conditions were recalculated accordingly. Statistical analysis (ANOVA with Tukey’s multiple comparison post test) was performed (ns not significant).
(TIF)

Figure S9 **Heat shock stimulates HSF1 heat shock granules, but CpdA does not.** A549 cells, starved for 48h in Optimem, were treated with solvent (Solv) for 60 minutes or CpdA (10µM) for 30 or 60 minutes. Alternatively, cells were heat-shocked (HS) at 43°C for 30 minutes. Via indirect immunofluorescence using an α-HSF1 Ab, endogenous HSF1 was visualized (green) and DAPI staining indicates the nuclei of the cells (blue). We also present an overlay and in the below panel, we digitally zoom in on one cell. White arrow heads indicate nuclear stress granules or foci of HSF1. This experiment is representative for 2 independent experiments.
(TIF)

Figure S10 **CpdA does not augment the HSF1 level, nor does it shift the band hight.** (A) A549 cells were treated with Solv, CpdA (10µM) or DEX (1µM) for 2, 4, 6 or 24 hours. Alternatively, cells were heat-shocked at 43°C for 2h, after which cells were left to recover at 37°C for 2h (HS+Rec). Total cell protein extracts were subjected to Western blot analysis detecting HSF1, with NF-κB p65 as a loading control. This image is representative for 2 independent experiments. (B) shows the averaged band densitometric analysis (ImageJ) of 2 independent HSF1 Western blot analyses. Specific HSF1 signal was corrected for sample loading. Solv was set as 1 to allow ratio comparisons. Statistical analysis (ANOVA with Tukey’s multiple comparison post test) was performed for selected pair wise comparisons (ns not significant).
(TIF)

Table S1 **Detailed siRNA information (Dharmacon, Thermo Fischer).**
(DOCX)

Table S2 **List of identified heat shock proteins and their corresponding peptides.** HEK293T cells were transfected with Flag-hGRα via calcium phosphate and following stimulation with either solvent (NI) or 10µM CpdA (CpdA), immunoprecipitated using Flag beads (plasmid and methodology described in [7]. Two identical set-ups were done in parallel, yet, using two different elution methods, via NH_4_OH, as indicated, or via the Flag peptide (FLAG, 100µg/ml). The identified heat shock proteins are listed and, per protein, the corresponding peptides (at 99% confidence settings) are shown (note that Mox indicates methionine-sulfoxide (oxidation) and Q<Pyr>indicates N-terminal pyroglutamic acid modification). The number of MS/MS spectra recorded per identified peptide is indicated.
(DOCX)

Materials and Methods S1 **All materials and methods, as described in the manuscript are also valid for the supporting information figures S1-S10 and tables S1 and S2.** Additional materials en methods to understand the supporting information figures and tables are added as a supporting file.
(DOCX)
