# Supplementary figures and images for: Compound A, a Selective Glucocorticoid Receptor Modulator, Enhances Heat Shock Protein Hsp70 Gene Promoter Activation
Source: PLoS One. 2013 Jul 30;8(7):e69115. doi: 10.1371/journal.pone.0069115 (PMC3728325; doi:10.1371/journal.pone.0069115)

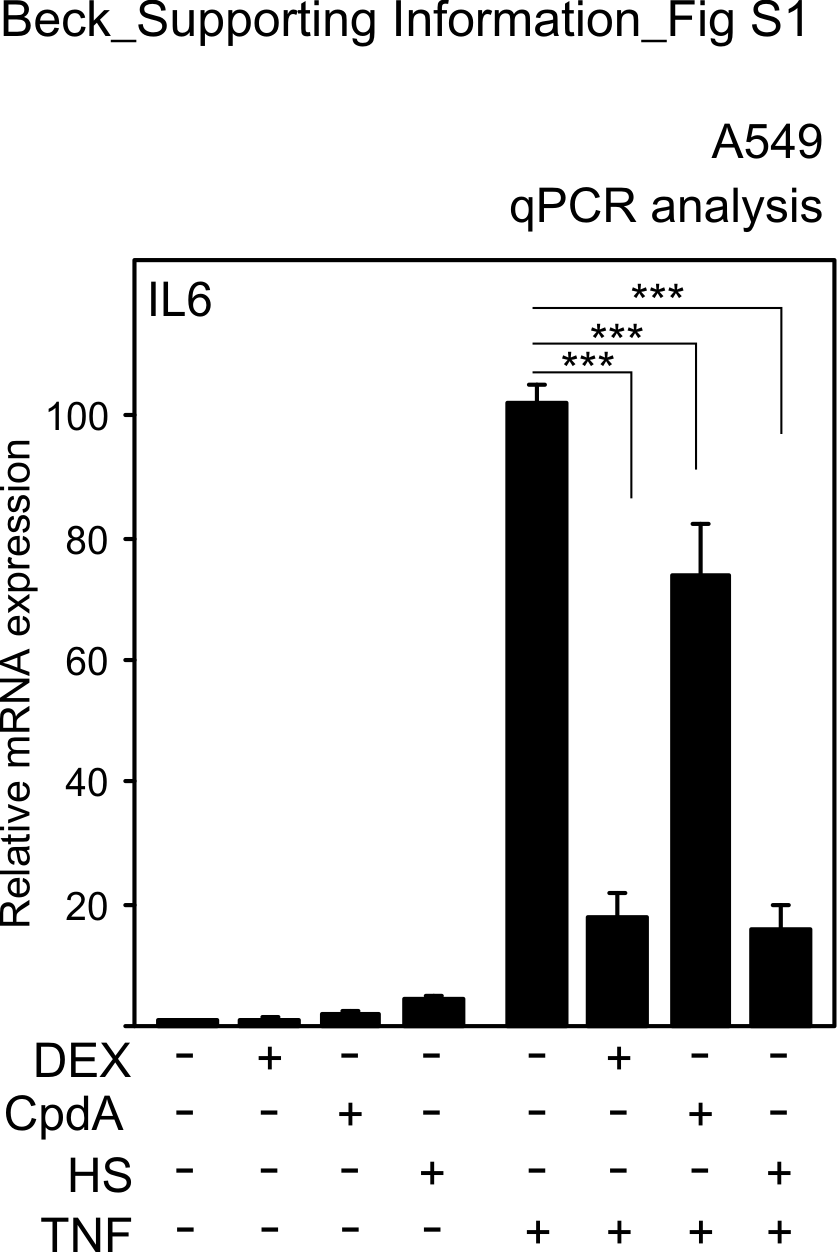

Supplement: Figure S1 — Both Compound A and heat shock diminish IL6 gene expression. (A) A549 cells, starved for 48h, were pretreated for 1.5h with solvent (Solv), DEX (1µM), CpdA (10µM) or subjected to heat shock treatment (1h at 43°C and 30′ recovery at 37°C), ensued with TNF (2000IU/ml) for 5.5h. Isolated total RNA was subjected to RT-qPCR assaying IL6 mRNA levels, normalized to cyclophilin household gene mRNA levels. The TNF condition was set at 100 and results were recalculated accordingly. These results are representative of 2 independent experiments. Statistical analysis (ANOVA and Tukey multiple comparison post test) were performed for selected pair-wise comparisons. (TIF) [file pone.0069115.s001.tif]

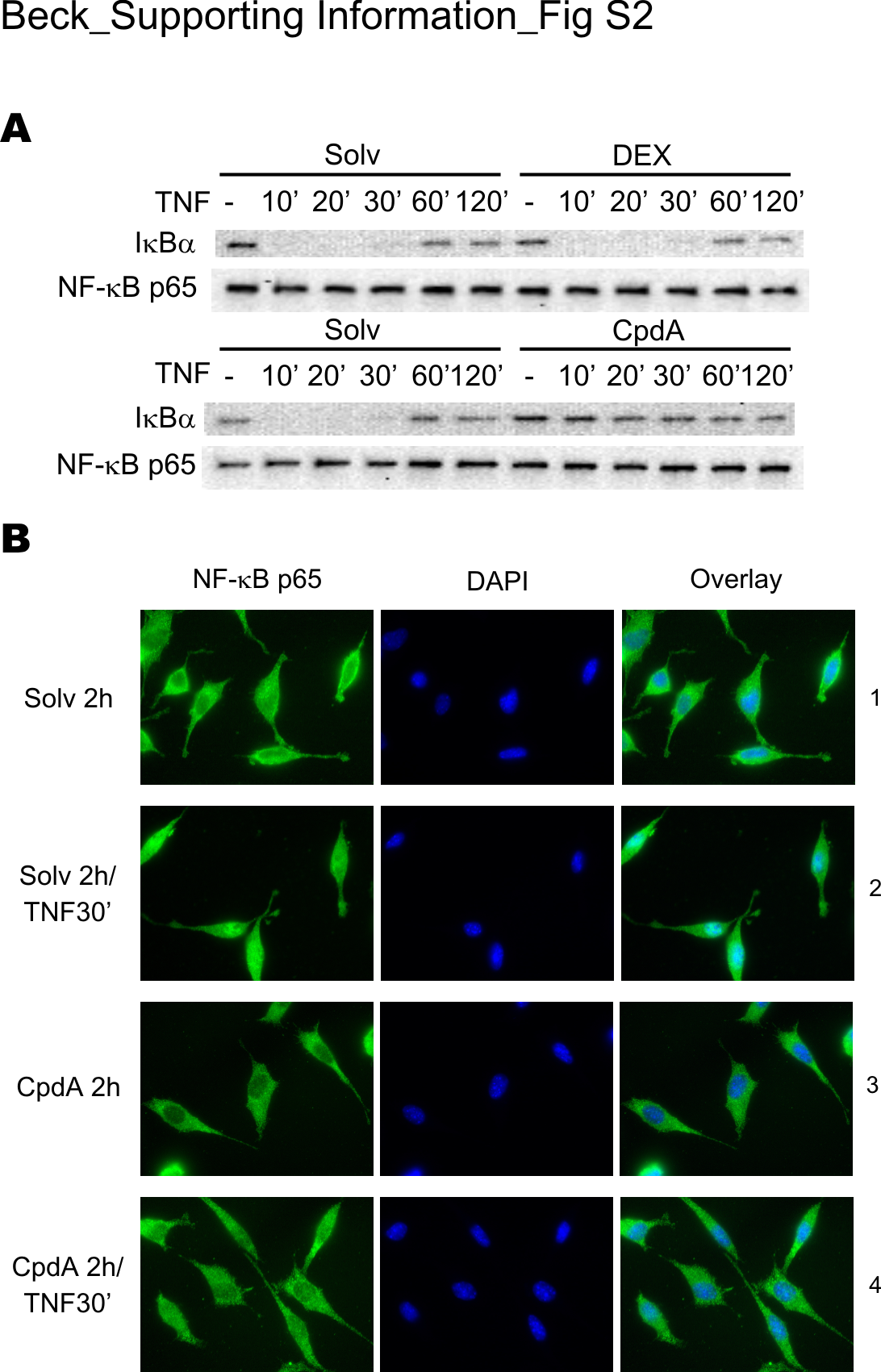

Supplement: Figure S2 — Compound A diminishes IκBα degradation and NF-κB translocation. (A) L929sA cells, starved for 48h, were pretreated for 2h with solvent (Solv), DEX (1µM) or CpdA (10µM), after which TNF (2000IU/ml) was added as indicated. Western blot analysis of total cells lysates detects IκBα protein, with NF-κB p65 as loading control. This figure is representative for 2 independent experiments. (B) L929sA cells, starved for 48h in Optimem, were pretreated for 2h with Solvent or CpdA (10µM). Subsequently, TNF (2000IU/ml) was added for 30′, where indicated. After washing, fixation, and permeabilization, indirect immunofluorescence detects endogenous NF-κB p65. DAPI staining indicates the nuclei. Additionally, we present overlays. (TIF) [file pone.0069115.s002.tif]

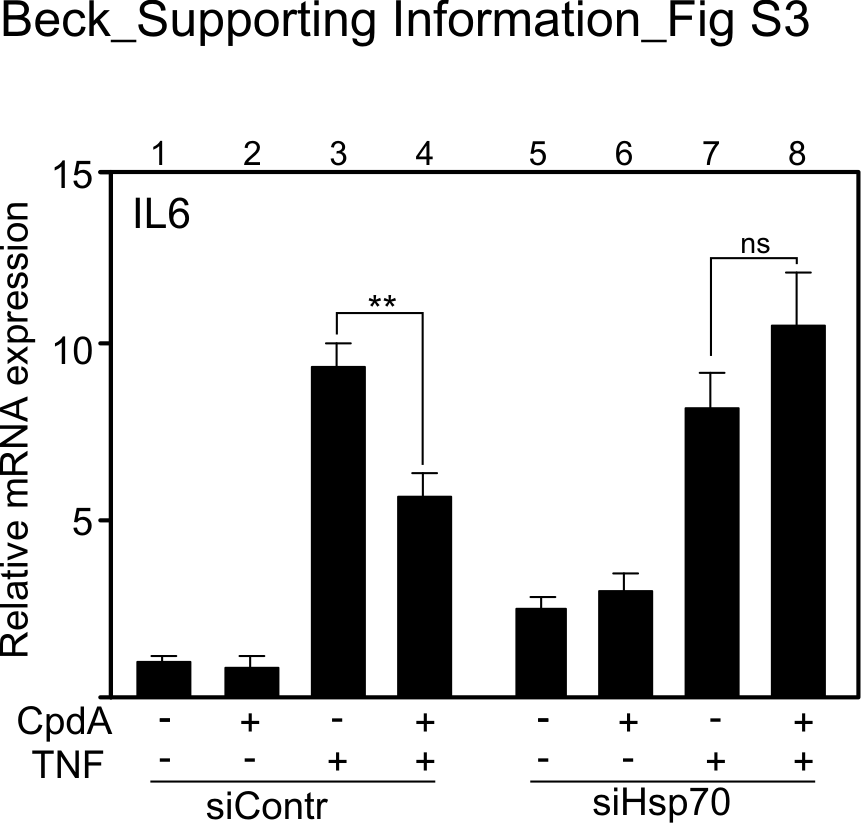

Supplement: Figure S3 — Hsp70 is required to allow the anti-inflammatory activity of Compound A. A549 cells were transfected with siControl or siRNA targeting HSPA1A and HSPA1B (siHsp70). 41h post transfection, cells were pretreated with Solv or CpdA (10µM) for 2h, after which ensued a 6h TNF (2000IU/ml) treatment. Total RNA extracts were prepared. Purified mRNA was subjected to RT-qPCR detecting IL6 gene expression levels and specific results were normalized to housekeeping controls cyclophilin and 28S. The condition Solv (siControl) was set as 1 to allow ratio comparisons. Statistical analysis (ANOVA with Tukey’s multiple comparison post test) was performed to show significant difference for selected pair wise comparisons (ns not significant; ** p<0.01). (TIF) [file pone.0069115.s003.tif]

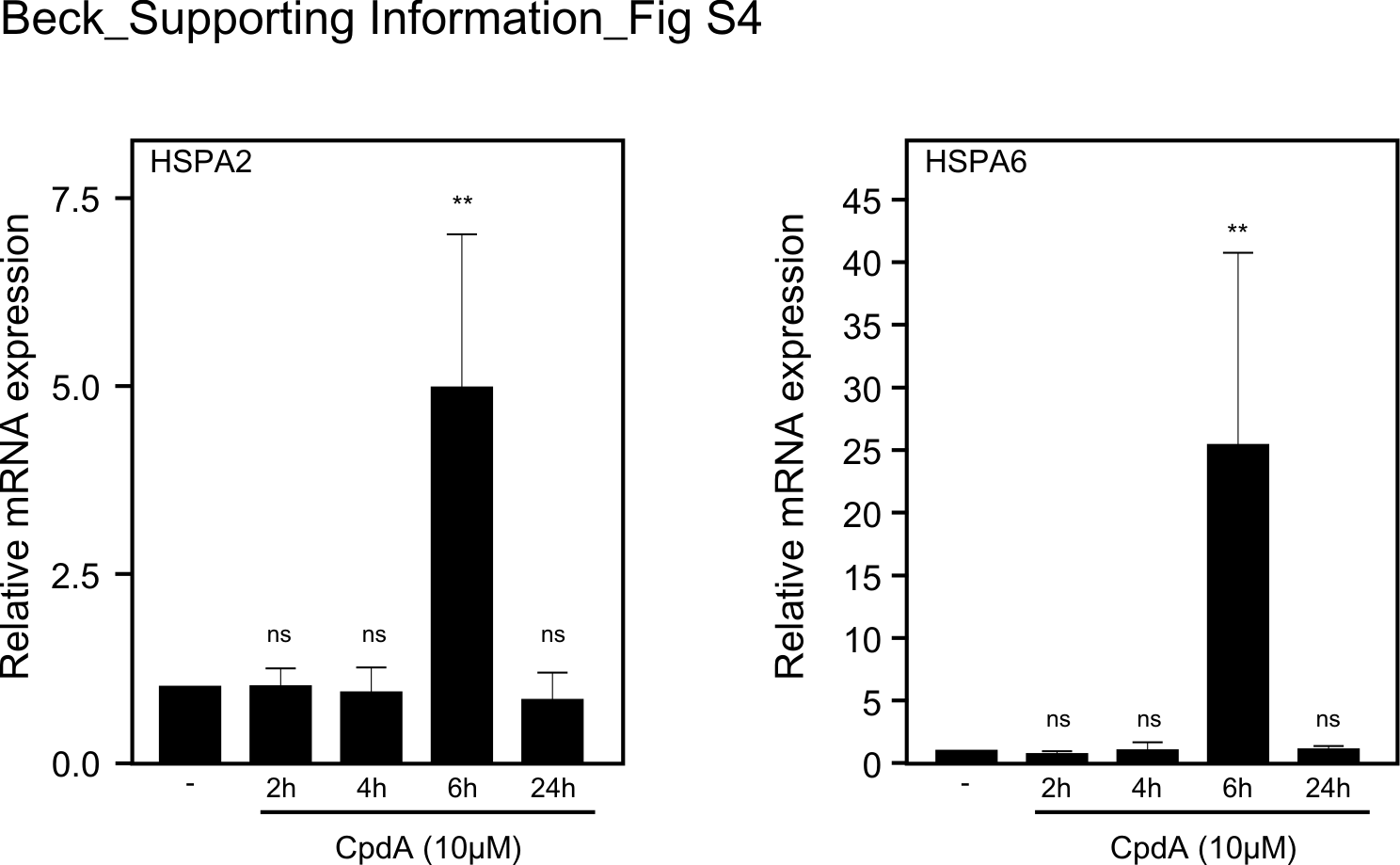

Supplement: Figure S4 — Compound A augments Hsp70 gene expression. A549 cells, were treated with solvent or CpdA 10µM for the indicated time period. Total cellular mRNA was subjected to RT-qPCR detecting gene expression levels for HSPA2 or HSPA6 (as indicated), normalized using housekeeping 36B4 and β-actin mRNA levels. The Solv condition was set as 1 and results recalculated accordingly. Statistical analysis (ANOVA with Tukey’s multiple comparison post test) was performed to compare with Solv (ns not significant; ** p<0.01). Three independent experiments with slightly varying time kinetics all show comparable results. (B) MCF-7 breast cancer cells (TIF) [file pone.0069115.s004.tif]

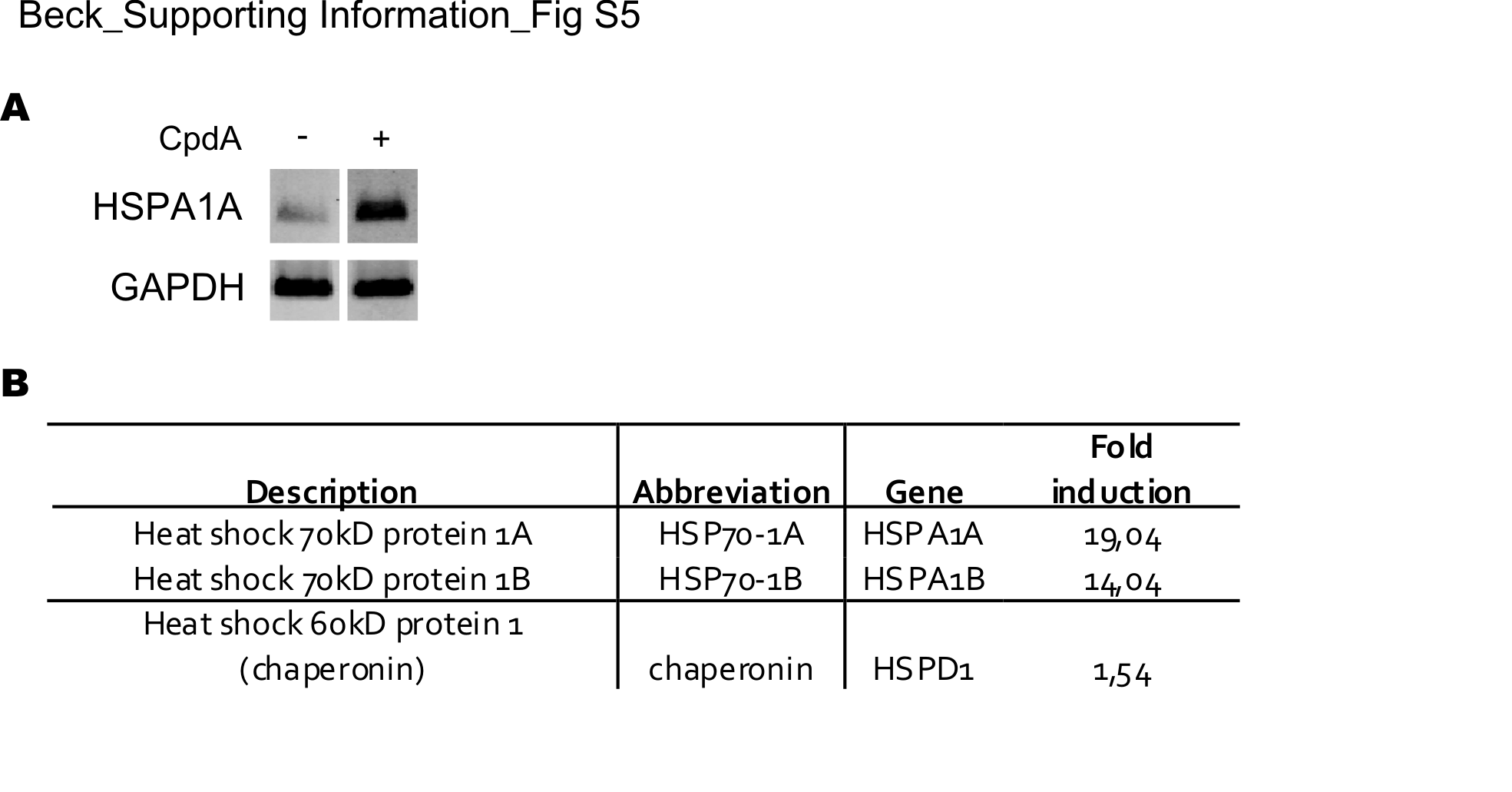

Supplement: Figure S5 — CpdA can elevate Hsp70 gene expression levels in MCF7 cells. (A) MCF7 cells were pretreated with solvent or CpdA (10µM) for 8 h. Total RNA was reverse transcribed and HSPA1A and housekeeping GAPDH mRNA levels were determined via semi-quantitative PCR visualized on a 2% agarose gels. The displayed bands were detected from one single gel. (B) MCF7 cells were assayed via the 'GEarray Q series Analysis with Human Stress and Toxicity pathway' (SABiosciences). Cells were treated with solvent or CpdA (10 µM) for 8h. Total RNA was isolated and reverse transcribed to hybridize labeled cDNA to Human Stress and Toxicity GEA array membranes. Results, visualized via Phospho-Imager, were quantified and controlled for by housekeeping genes. Effects of CpdA are presented as 'fold induction'. (TIF) [file pone.0069115.s005.tif]

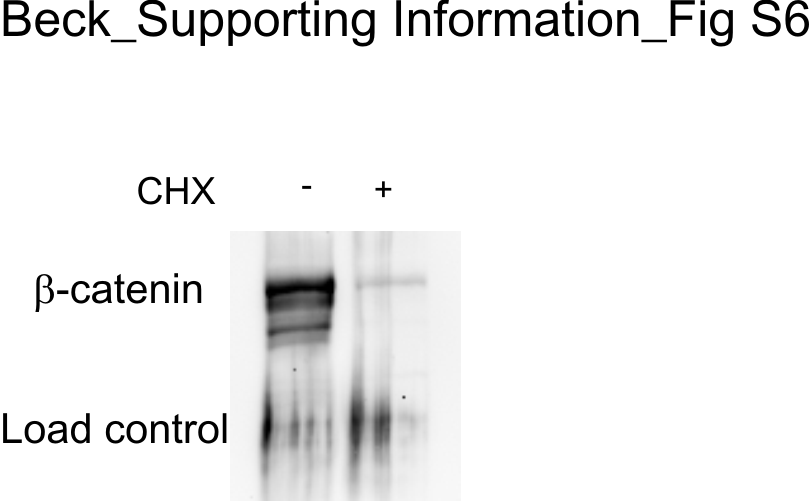

Supplement: Figure S6 — Control of CHX functionality. A549 cells, starved for 48h, were left untreated or were treated for 7h with cycloheximide (CHX) (20µg/ml). Total cell protein extracts were subjected to Western blot analysis detecting β-catenin and an aspecific band serves as a loading control. (TIF) [file pone.0069115.s006.tif]

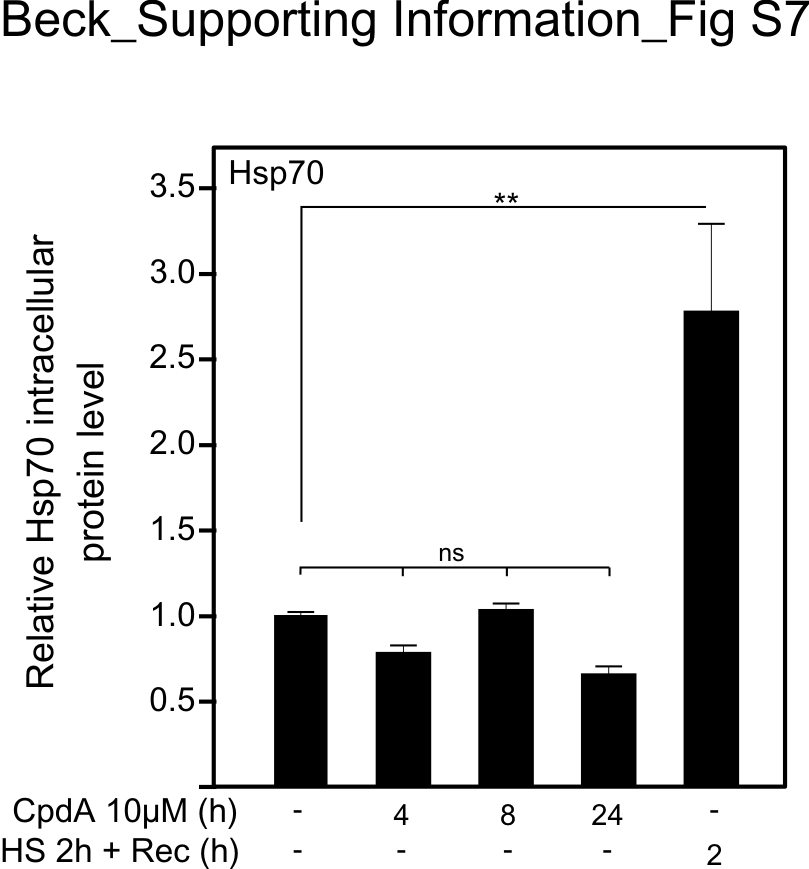

Supplement: Figure S7 — CpdA does not elevate the Hsp70 protein level in L929sA cells. L929sA cells were treated with solvent or CpdA (10µM) for 4h,8h or 24h or heat-shocked at 43°C for 2h, after which cells were left to recover at 37°C for 2h (HS+Rec). Total cell protein lysates were analyzed via Hsp70 ELISA. Statistical analysis (ANOVA with Tukey’s multiple comparison post test) was performed for selected pair-wise comparisons (ns not significant; **p<0.01). This figure represents averaged data of 2 independent experiments. (TIF) [file pone.0069115.s007.tif]

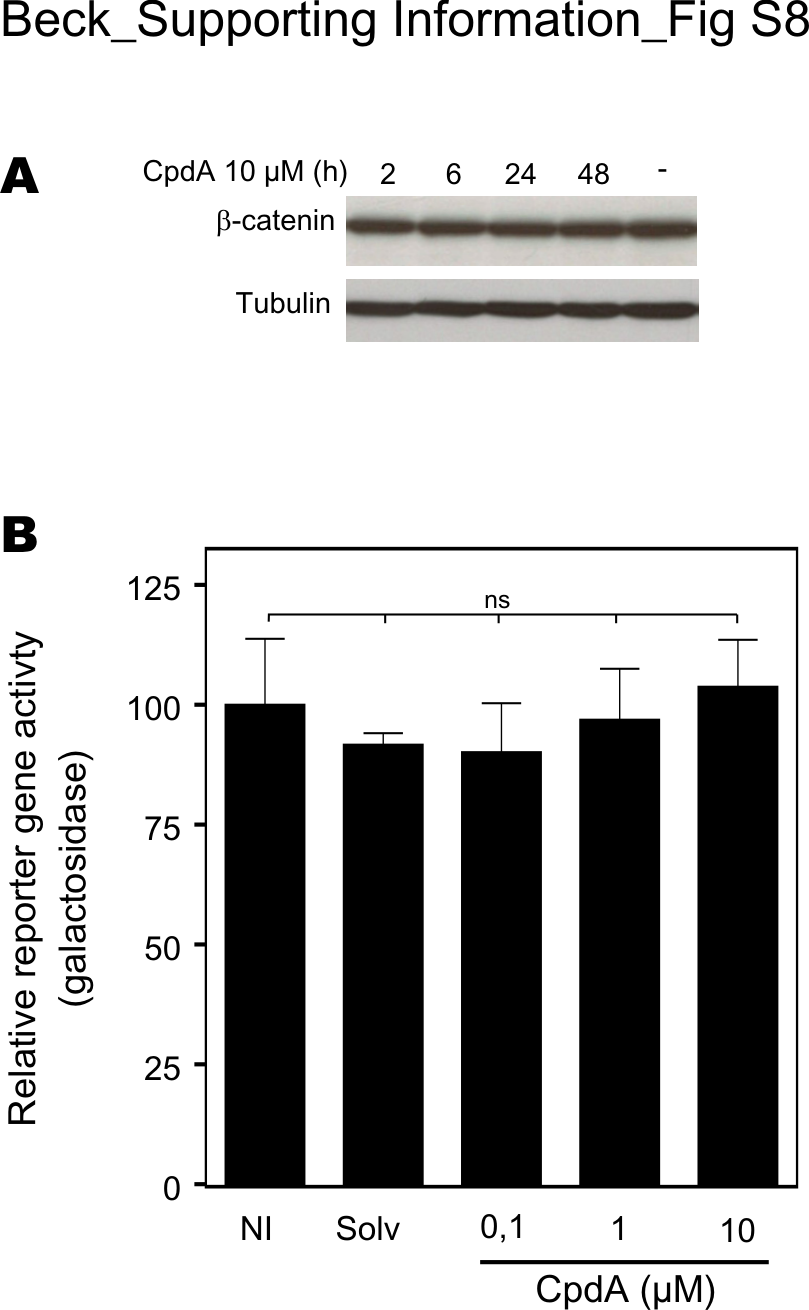

Supplement: Figure S8 — Compound A does not block translation. (A) PC-3 cells were starved for 48h in 0% DMEM, after which these cells were treated with solvent for 48h or Compound A (CpdA) (10µM) for 2h, 6h, 24h or 48h. Total cell protein extracts were subjected to Western blot analysis detecting β-catenin. Tubulin detection served as a loading control. (B) L929sA cells, stably transfected with p(IL6κB)350hu.IL6P-luc+, were left untreated (NI), or were treated with solvent (Solv), or CpdA (0.1µM, 1µM or 10µM) for 8h. The relative activity of the constitutively expressed galactosidase (β-gal) controls were presented as relative reporter gene activity with the condition Solv set at 100. All other conditions were recalculated accordingly. Statistical analysis (ANOVA with Tukey’s multiple comparison post test) was performed (ns not significant). (TIF) [file pone.0069115.s008.tif]

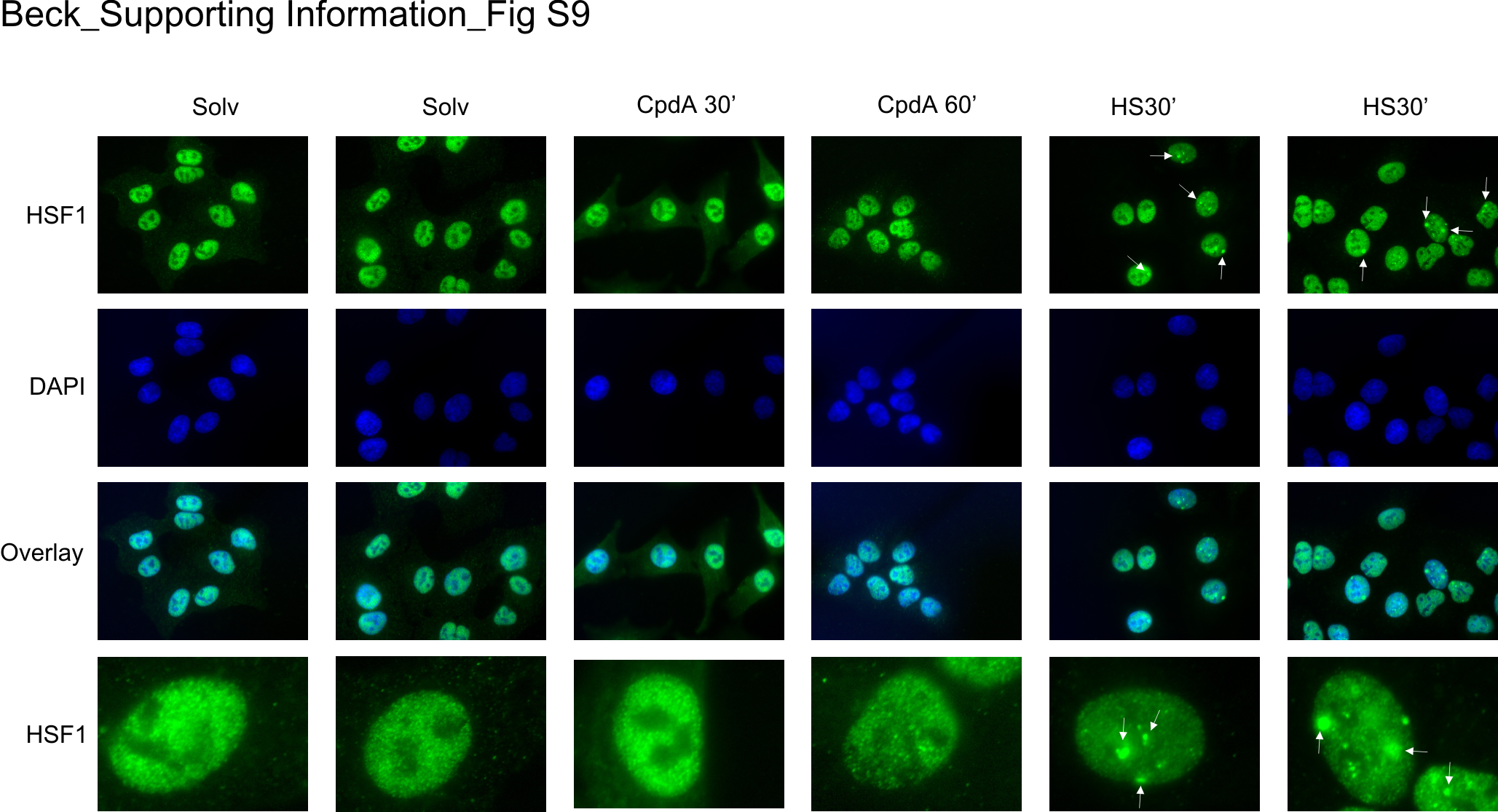

Supplement: Figure S9 — Heat shock stimulates HSF1 heat shock granules, but CpdA does not. A549 cells, starved for 48h in Optimem, were treated with solvent (Solv) for 60 minutes or CpdA (10µM) for 30 or 60 minutes. Alternatively, cells were heat-shocked (HS) at 43°C for 30 minutes. Via indirect immunofluorescence using an α-HSF1 Ab, endogenous HSF1 was visualized (green) and DAPI staining indicates the nuclei of the cells (blue). We also present an overlay and in the below panel, we digitally zoom in on one cell. White arrow heads indicate nuclear stress granules or foci of HSF1. This experiment is representative for 2 independent experiments. (TIF) [file pone.0069115.s009.tif]

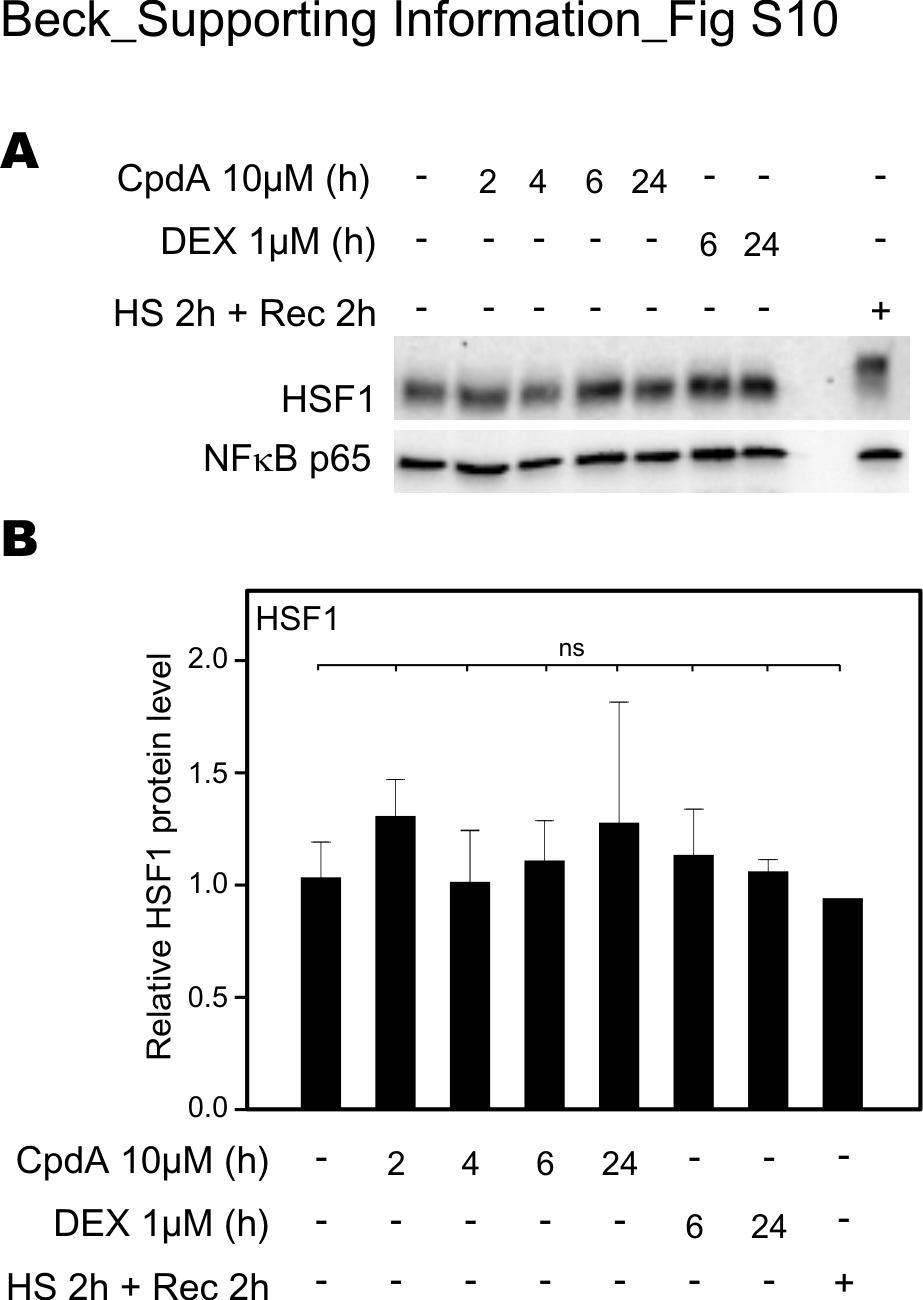

Supplement: Figure S10 — CpdA does not augment the HSF1 level, nor does it shift the band hight. (A) A549 cells were treated with Solv, CpdA (10µM) or DEX (1µM) for 2, 4, 6 or 24 hours. Alternatively, cells were heat-shocked at 43°C for 2h, after which cells were left to recover at 37°C for 2h (HS+Rec). Total cell protein extracts were subjected to Western blot analysis detecting HSF1, with NF-κB p65 as a loading control. This image is representative for 2 independent experiments. (B) shows the averaged band densitometric analysis (ImageJ) of 2 independent HSF1 Western blot analyses. Specific HSF1 signal was corrected for sample loading. Solv was set as 1 to allow ratio comparisons. Statistical analysis (ANOVA with Tukey’s multiple comparison post test) was performed for selected pair wise comparisons (ns not significant). (TIF) [file pone.0069115.s010.tif]
