# Supplementary material for: Compound A, a Selective Glucocorticoid Receptor Modulator, Enhances Heat Shock Protein Hsp70 Gene Promoter Activation
Source: PLoS One. 2013 Jul 30;8(7):e69115. doi: 10.1371/journal.pone.0069115 (PMC3728325; doi:10.1371/journal.pone.0069115)
Supplement: Table S1 — Detailed siRNA information (Dharmacon, Thermo Fischer). (DOCX) [file pone.0069115.s011.docx]

**Supporting information Table S1. Detailed siRNA information (Dharmacon, Thermo Fischer)**

| **Product Name** | **Pool Catalog #** | **Individual Catalog #** | **Target sequence** |
| --- | --- | --- | --- |
| siCONTROL Non-Targeting siRNA Pool | D-001206-13 | D-001210-01 | UAGCGACUAAACACAUCAA |
|  |  | D-001210-02 | UAAGGCUAUGAAGAGAUAC |
|  |  | D-001210-03 | AUGUAUUGGCCUGUAUUAG |
|  |  | D-001210-04 | AUGAACGUGAAUUGCUCAA |
| siGENOME SMARTpool human HSPA1A | M-005168-01 | D-005168-01 | GAGAUCGACUCCCUGUUUG |
|  |  | D-005168-03 | GAUCAACGACGGAGACAAG |
|  |  | D-005168-04 | GCGCUGAACCCGCAGAACA |
|  |  | D-005168-05 | GCUCCGACCUGUUCCGAAG |
| siGENOME SMARTpool Human HSPA1B | M-003501-03 | D-003501-03 | GAUCAACGACGGAGACAAG |
|  |  | D-003501-05 | GAGAUCGACUCCCUGUUUG |
|  |  | D-003501-08 | AGAGGGCCAUGACGAAAGA |
|  |  | D-003501-09 | GCUCCGACCUGUUCCGAAG |

| **Product Name** | **Pool Catalog #** | **Individual Catalog #** | **Sequences** |  |
| --- | --- | --- | --- | --- |
| siGENOME SMARTpool Human NR3C1 | M-003424-02 | D-003424-02 | GAUAAGACCAUGAGUAUUGUU | sense |
| siGENOME siGENOME SMARTpool duplex |  |  | 5'-PCAAUACUCAUGGUCUUAUCUU | antisense |
|  |  | D-003424-04 | GGACAGAUGUACCACUAUGUU | sense |
|  |  |  | 5'-PCAUAGUGGUACAUCUGUCCUU | antisense |
|  |  | D-003424-05 | GAGGACAGAUGUACCACUAUU | sense |
|  |  |  | 5'-PAUGUGGUACAUCUGUCCUCUU | antisense |
|  |  | D-003424-06 | GAACUUCCCUGGUCGAACAUU | sense |
|  |  |  | 5'-PUGUUCGACCAGGGAAGUUCUU | antisense |
