# Supplementary material for: Compound A, a Selective Glucocorticoid Receptor Modulator, Enhances Heat Shock Protein Hsp70 Gene Promoter Activation
Source: PLoS One. 2013 Jul 30;8(7):e69115. doi: 10.1371/journal.pone.0069115 (PMC3728325; doi:10.1371/journal.pone.0069115)
Supplement: Table S2 — List of identified heat shock proteins and their corresponding peptides. HEK293T cells were transfected with Flag-hGRα via calcium phosphate and following stimulation with either solvent (NI) or 10µM CpdA (CpdA), immunoprecipitated using Flag beads (plasmid and methodology described in [7]. Two identical set-ups were done in parallel, yet, using two different elution methods, via NH4OH, as indicated, or via the Flag peptide (FLAG, 100µg/ml). The identified heat shock proteins are listed and, per protein, the corresponding peptides (at 99% confidence settings) are shown (note that Mox indicates methionine-sulfoxide (oxidation) and Qindicates N-terminal pyroglutamic acid modification). The number of MS/MS spectra recorded per identified peptide is indicated. (DOCX) [file pone.0069115.s012.docx]

**Supporting information Table S2. List of identified heat shock proteins and their corresponding peptides.**

HEK293T cells were transfected with Flag-hGRα via calcium phosphate and following stimulation with either solvent (NI) or 10μM CpdA (CpdA), immunoprecipitated using Flag beads (plasmid and methodology described in [7]. Two identical set-ups were done in parallel, yet, using two different elution methods, via NH_4_OH, as indicated, or via the Flag peptide (FLAG, 100μg/ml). The identified heat shock proteins are listed and, per protein, the corresponding peptides (at 99% confidence settings) are shown (note that Mox indicates methionine-sulfoxide (oxidation) and Q<Pyr> indicates N-terminal pyroglutamic acid modification). The number of MS/MS spectra recorded per identified peptide is indicated.

| **Identified proteins** | **Identified peptides** | **Start** | **End** | **Spectra** | **Run** |
| --- | --- | --- | --- | --- | --- |
| Heat shock 70 kDa protein 1A/1B (HSP71_HUMAN) | VEIIANDQGNR | 26 | 36 | 2 | CpdA (FLAG) |
|  | TTPSYVAFTDTER | 37 | 49 | 3 | CpdA (FLAG) |
|  | LIGDAAKNQVALNPQNTVFDAK | 50 | 71 | 1 | CpdA (FLAG) |
|  | NQVALNPQNTVFDAK | 57 | 71 | 2 | CpdA (FLAG) |
|  | KFGDPVVQSDMK | 77 | 88 | 2 | CpdA (FLAG) |
|  | FGDPVVQSDM<Mox>K | 78 | 88 | 1 | CpdA (FLAG) |
|  | FGDPVVQSDMK | 78 | 88 | 2 | CpdA (FLAG) |
|  | HWPFQVINDGDKPK | 89 | 102 | 3 | CpdA (FLAG) |
|  | VQVSYKGETK | 103 | 112 | 1 | CpdA (FLAG) |
|  | AFYPEEISSM<Mox>VLTK | 113 | 126 | 2 | CpdA (FLAG) |
|  | AFYPEEISSMVLTK | 113 | 126 | 2 | CpdA (FLAG) |
|  | M<Mox>KEIAEAYLGYPVTNAVITVPAYFNDSQR | 127 | 155 | 1 | CpdA (FLAG) |
|  | MKEIAEAYLGYPVTNAVITVPAYFNDSQR | 127 | 155 | 1 | CpdA (FLAG) |
|  | EIAEAYLGYPVTNAVITVPAYFNDSQR | 129 | 155 | 1 | CpdA (FLAG) |
|  | QATKDAGVIAGLNVLR | 156 | 171 | 1 | CpdA (FLAG) |
|  | DAGVIAGLNVLR | 160 | 171 | 2 | CpdA (FLAG) |
|  | IINEPTAAAIAYGLDR | 172 | 187 | 3 | CpdA (FLAG) |
|  | ATAGDTHLGGEDFDNR | 221 | 236 | 2 | CpdA (FLAG) |
|  | LVNHFVEEFK | 237 | 246 | 1 | CpdA (FLAG) |
|  | LVNHFVEEFKR | 237 | 247 | 3 | CpdA (FLAG) |
|  | LDKAQIHDLVLVGGSTR | 326 | 342 | 1 | CpdA (FLAG) |
|  | AQIHDLVLVGGSTR | 329 | 342 | 1 | CpdA (FLAG) |
|  | LLQDFFNGR | 349 | 357 | 1 | CpdA (FLAG) |
|  | SINPDEAVAYGAAVQAAILM<Mox>GDK | 362 | 384 | 1 | CpdA (FLAG) |
|  | SINPDEAVAYGAAVQAAILMGDK | 362 | 384 | 1 | CpdA (FLAG) |
|  | SENVQDLLLLDVAPLSLGLETAGGVMTALIK | 385 | 415 | 1 | CpdA (FLAG) |
|  | QTQIFTTYSDNQPGVLIQVYEGER | 424 | 447 | 2 | CpdA (FLAG) |
|  | FELSGIPPAPR | 459 | 469 | 1 | CpdA (FLAG) |
|  | LSKEEIER | 510 | 517 | 1 | CpdA (FLAG) |
|  | NALESYAFNM<Mox>K | 540 | 550 | 1 | CpdA (FLAG) |
|  | NALESYAFNMK | 540 | 550 | 3 | CpdA (FLAG) |
|  | GGSGSGPTIEEVD | 629 | 641 | 1 | CpdA (FLAG) |
| Heat shock 70 kDa protein 1A/1B (HSP71_HUMAN) | VEIIANDQGNR | 26 | 36 | 2 | CpdA (NH_4_OH) |
|  | TTPSYVAFTDTER | 37 | 49 | 1 | CpdA (NH_4_OH) |
|  | NQVALNPQNTVFDAK | 57 | 71 | 1 | CpdA (NH_4_OH) |
|  | KFGDPVVQSDMK | 77 | 88 | 2 | CpdA (NH_4_OH) |
|  | FGDPVVQSDM<Mox>K | 78 | 88 | 1 | CpdA (NH_4_OH) |
|  | FGDPVVQSDMK | 78 | 88 | 1 | CpdA (NH_4_OH) |
|  | HWPFQVINDGDKPK | 89 | 102 | 2 | CpdA (NH_4_OH) |
|  | AFYPEEISSMVLTK | 113 | 126 | 2 | CpdA (NH_4_OH) |
|  | MKEIAEAYLGYPVTNAVITVPAYFNDSQR | 127 | 155 | 1 | CpdA (NH_4_OH) |
|  | EIAEAYLGYPVTNAVITVPAYFNDSQR | 129 | 155 | 2 | CpdA (NH_4_OH) |
|  | DAGVIAGLNVLR | 160 | 171 | 1 | CpdA (NH_4_OH) |
|  | IINEPTAAAIAYGLDR | 172 | 187 | 2 | CpdA (NH_4_OH) |
|  | ATAGDTHLGGEDFDNR | 221 | 236 | 2 | CpdA (NH_4_OH) |
|  | LVNHFVEEFK | 237 | 246 | 1 | CpdA (NH_4_OH) |
|  | LVNHFVEEFKR | 237 | 247 | 2 | CpdA (NH_4_OH) |
|  | TLSSSTQASLEIDSLFEGIDFYTSITR | 273 | 299 | 3 | CpdA (NH_4_OH) |
|  | AQIHDLVLVGGSTR | 329 | 342 | 2 | CpdA (NH_4_OH) |
|  | LLQDFFNGR | 349 | 357 | 1 | CpdA (NH_4_OH) |
|  | SINPDEAVAYGAAVQAAILM<Mox>GDK | 362 | 384 | 1 | CpdA (NH_4_OH) |
|  | SINPDEAVAYGAAVQAAILMGDK | 362 | 384 | 4 | CpdA (NH_4_OH) |
|  | SENVQDLLLLDVAPLSLGLETAGGVMTALIK | 385 | 415 | 4 | CpdA (NH_4_OH) |
|  | Q<Pyr>TQIFTTYSDNQPGVLIQVYEGER | 424 | 447 | 1 | CpdA (NH_4_OH) |
|  | QTQIFTTYSDNQPGVLIQVYEGER | 424 | 447 | 1 | CpdA (NH_4_OH) |
|  | GVPQIEVTFDIDANGILNVTATDK | 470 | 493 | 4 | CpdA (NH_4_OH) |
|  | LSKEEIER | 510 | 517 | 1 | CpdA (NH_4_OH) |
|  | YKAEDEVQR | 525 | 533 | 1 | CpdA (NH_4_OH) |
|  | NALESYAFNMK | 540 | 550 | 1 | CpdA (NH_4_OH) |
|  | GGSGSGPTIEEVD | 629 | 641 | 1 | CpdA (NH_4_OH) |
| Heat shock 70 kDa protein 1A/1B (HSP71_HUMAN) | VEIIANDQGNR | 26 | 36 | 2 | NI (FLAG) |
|  | TTPSYVAFTDTER | 37 | 49 | 3 | NI (FLAG) |
|  | NQVALNPQNTVFDAK | 57 | 71 | 2 | NI (FLAG) |
|  | KFGDPVVQSDMK | 77 | 88 | 1 | NI (FLAG) |
|  | FGDPVVQSDM<Mox>K | 78 | 88 | 1 | NI (FLAG) |
|  | FGDPVVQSDMK | 78 | 88 | 1 | NI (FLAG) |
|  | HWPFQVINDGDKPK | 89 | 102 | 3 | NI (FLAG) |
|  | VQVSYKGETK | 103 | 112 | 1 | NI (FLAG) |
|  | AFYPEEISSM<Mox>VLTK | 113 | 126 | 2 | NI (FLAG) |
|  | AFYPEEISSMVLTK | 113 | 126 | 2 | NI (FLAG) |
|  | MKEIAEAYLGYPVTNAVITVPAYFNDSQR | 127 | 155 | 2 | NI (FLAG) |
|  | EIAEAYLGYPVTNAVITVPAYFNDSQR | 129 | 155 | 1 | NI (FLAG) |
|  | DAGVIAGLNVLR | 160 | 171 | 2 | NI (FLAG) |
|  | IINEPTAAAIAYGLDR | 172 | 187 | 4 | NI (FLAG) |
|  | NVLIFDLGGGTFDVSILTIDDGIFEVK | 194 | 220 | 2 | NI (FLAG) |
|  | ATAGDTHLGGEDFDNR | 221 | 236 | 2 | NI (FLAG) |
|  | LVNHFVEEFK | 237 | 246 | 2 | NI (FLAG) |
|  | LVNHFVEEFKR | 237 | 247 | 3 | NI (FLAG) |
|  | LDKAQIHDLVLVGGSTR | 326 | 342 | 1 | NI (FLAG) |
|  | AQIHDLVLVGGSTR | 329 | 342 | 2 | NI (FLAG) |
|  | LLQDFFNGR | 349 | 357 | 1 | NI (FLAG) |
|  | SINPDEAVAYGAAVQAAILM<Mox>GDK | 362 | 384 | 1 | NI (FLAG) |
|  | SINPDEAVAYGAAVQAAILMGDK | 362 | 384 | 2 | NI (FLAG) |
|  | SENVQDLLLLDVAPLSLGLETAGGVMTALIK | 385 | 415 | 1 | NI (FLAG) |
|  | QTQIFTTYSDNQPGVLIQVYEGER | 424 | 447 | 3 | NI (FLAG) |
|  | LSKEEIER | 510 | 517 | 1 | NI (FLAG) |
|  | NALESYAFNM<Mox>K | 540 | 550 | 1 | NI (FLAG) |
|  | NALESYAFNMK | 540 | 550 | 2 | NI (FLAG) |
|  | GGSGSGPTIEEVD | 629 | 641 | 1 | NI (FLAG) |
| Heat shock 70 kDa protein 1A/1B (HSP71_HUMAN) | VEIIANDQGNR | 26 | 36 | 1 | NI (NH_4_OH) |
|  | TTPSYVAFTDTER | 37 | 49 | 1 | NI (NH_4_OH)) |
|  | NQVALNPQNTVFDAK | 57 | 71 | 1 | NI (NH_4_OH) |
|  | KFGDPVVQSDMK | 77 | 88 | 2 | NI (NH_4_OH) |
|  | FGDPVVQSDMK | 78 | 88 | 1 | NI (NH_4_OH) |
|  | HWPFQVINDGDKPK | 89 | 102 | 1 | NI (NH_4_OH) |
|  | AFYPEEISSM<Mox>VLTK | 113 | 126 | 1 | NI (NH_4_OH) |
|  | AFYPEEISSMVLTK | 113 | 126 | 2 | NI (NH_4_OH) |
|  | DAGVIAGLNVLR | 160 | 171 | 2 | NI (NH_4_OH)) |
|  | IINEPTAAAIAYGLDR | 172 | 187 | 2 | NI (NH_4_OH) |
|  | NVLIFDLGGGTFDVSILTIDDGIFEVK | 194 | 220 | 3 | NI (NH_4_OH) |
|  | ATAGDTHLGGEDFDNR | 221 | 236 | 2 | NI (NH_4_OH) |
|  | LVNHFVEEFK | 237 | 246 | 1 | NI (NH_4_OH) |
|  | LVNHFVEEFKR | 237 | 247 | 2 | NI (NH_4_OH) |
|  | TLSSSTQASLEIDSLFEGIDFYTSITR | 273 | 299 | 1 | NI (NH_4_OH) |
|  | AQIHDLVLVGGSTR | 329 | 342 | 2 | NI (NH_4_OH)) |
|  | LLQDFFNGR | 349 | 357 | 1 | NI (NH_4_OH) |
|  | SINPDEAVAYGAAVQAAILM<Mox>GDK | 362 | 384 | 1 | NI (NH_4_OH)) |
|  | SINPDEAVAYGAAVQAAILMGDK | 362 | 384 | 3 | NI (NH_4_OH) |
|  | SENVQDLLLLDVAPLSLGLETAGGVM<Mox>TALIK | 385 | 415 | 2 | NI (NH_4_OH) |
|  | SENVQDLLLLDVAPLSLGLETAGGVMTALIK | 385 | 415 | 4 | NI (NH_4_OH) |
|  | QTQIFTTYSDNQPGVLIQVYEGER | 424 | 447 | 1 | NI (NH_4_OH) |
|  | GVPQIEVTFDIDANGILNVTATDK | 470 | 493 | 9 | NI (NH_4_OH) |
|  | LSKEEIER | 510 | 517 | 1 | NI (NH_4_OH) |
|  | NALESYAFNM<Mox>K | 540 | 550 | 1 | NI (NH_4_OH)) |
|  | NALESYAFNMK | 540 | 550 | 1 | NI (NH_4_OH) |
|  | GGSGSGPTIEEVD | 629 | 641 | 1 | NI (NH_4_OH) |
| Heat shock cognate 71 kDa protein (HSP7C_HUMAN) | NQVAM<Mox>NPTNTVFDAK | 57 | 71 | 1 | CpdA (FLAG) |
|  | NQVAMNPTNTVFDAK | 57 | 71 | 2 | CpdA (FLAG) |
|  | RFDDAVVQSDM<Mox>K | 77 | 88 | 2 | CpdA (FLAG) |
|  | RFDDAVVQSDMK | 77 | 88 | 2 | CpdA (FLAG) |
|  | FDDAVVQSDM<Mox>K | 78 | 88 | 1 | CpdA (FLAG) |
|  | FDDAVVQSDMK | 78 | 88 | 1 | CpdA (FLAG) |
|  | HWPFM<Mox>VVNDAGRPK | 89 | 102 | 1 | CpdA (FLAG) |
|  | HWPFMVVNDAGR | 89 | 100 | 1 | CpdA (FLAG) |
|  | HWPFMVVNDAGRPK | 89 | 102 | 2 | CpdA (FLAG) |
|  | VQVEYKGETK | 103 | 112 | 1 | CpdA (FLAG) |
|  | SFYPEEVSSM<Mox>VLTK | 113 | 126 | 1 | CpdA (FLAG) |
|  | SFYPEEVSSMVLTK | 113 | 126 | 2 | CpdA (FLAG) |
|  | M<Mox>KEIAEAYLGK | 127 | 137 | 1 | CpdA (FLAG) |
|  | MKEIAEAYLGK | 127 | 137 | 2 | CpdA (FLAG) |
|  | EIAEAYLGK | 129 | 137 | 1 | CpdA (FLAG) |
|  | TVTNAVVTVPAYFNDSQR | 138 | 155 | 2 | CpdA (FLAG) |
|  | QATKDAGTIAGLNVLR | 156 | 171 | 1 | CpdA (FLAG) |
|  | DAGTIAGLNVLR | 160 | 171 | 2 | CpdA (FLAG) |
|  | NVLIFDLGGGTFDVSILTIEDGIFEVK | 194 | 220 | 1 | CpdA (FLAG) |
|  | STAGDTHLGGEDFDNR | 221 | 236 | 2 | CpdA (FLAG) |
|  | MVNHFIAEFK | 237 | 246 | 1 | CpdA (FLAG) |
|  | ARFEELNADLFR | 300 | 311 | 1 | CpdA (FLAG) |
|  | FEELNADLFR | 302 | 311 | 1 | CpdA (FLAG) |
|  | LDKSQIHDIVLVGGSTR | 326 | 342 | 2 | CpdA (FLAG) |
|  | SQIHDIVLVGGSTR | 329 | 342 | 2 | CpdA (FLAG) |
|  | LLQDFFNGK | 349 | 357 | 1 | CpdA (FLAG) |
|  | SINPDEAVAYGAAVQAAILSGDK | 362 | 384 | 1 | CpdA (FLAG) |
|  | QTQTFTTYSDNQPGVLIQVYEGER | 424 | 447 | 1 | CpdA (FLAG) |
|  | NSLESYAFNM<Mox>K | 540 | 550 | 1 | CpdA (FLAG) |
|  | NSLESYAFNMK | 540 | 550 | 2 | CpdA (FLAG) |
|  | NQTAEKEEFEHQQK | 584 | 597 | 2 | CpdA (FLAG) |
|  | LYQSAGGMPGGMPGGFPGGGAPPSGGASSGPTIEEVD | 610 | 646 | 1 | CpdA (FLAG) |
| Heat shock cognate 71 kDa protein (HSP7C_HUMAN) | NQVAM<Mox>NPTNTVFDAK | 57 | 71 | 1 | CpdA (NH_4_OH) |
|  | NQVAMNPTNTVFDAK | 57 | 71 | 1 | CpdA (NH_4_OH) |
|  | RFDDAVVQSDM<Mox>K | 77 | 88 | 1 | CpdA (NH_4_OH) |
|  | RFDDAVVQSDMK | 77 | 88 | 2 | CpdA (NH_4_OH) |
|  | FDDAVVQSDM<Mox>K | 78 | 88 | 1 | CpdA (NH_4_OH) |
|  | FDDAVVQSDMK | 78 | 88 | 1 | CpdA (NH_4_OH) |
|  | HWPFMVVNDAGR | 89 | 100 | 1 | CpdA (NH_4_OH) |
|  | HWPFMVVNDAGRPK | 89 | 102 | 1 | CpdA (NH_4_OH) |
|  | VQVEYKGETK | 103 | 112 | 1 | CpdA (NH_4_OH) |
|  | SFYPEEVSSMVLTK | 113 | 126 | 2 | CpdA (NH_4_OH) |
|  | MKEIAEAYLGK | 127 | 137 | 2 | CpdA (NH_4_OH) |
|  | EIAEAYLGK | 129 | 137 | 1 | CpdA (NH_4_OH) |
|  | TVTNAVVTVPAYFNDSQR | 138 | 155 | 2 | CpdA (NH_4_OH) |
|  | DAGTIAGLNVLR | 160 | 171 | 1 | CpdA (NH_4_OH) |
|  | NVLIFDLGGGTFDVSILTIEDGIFEVK | 194 | 220 | 1 | CpdA (NH_4_OH) |
|  | STAGDTHLGGEDFDNR | 221 | 236 | 2 | CpdA (NH_4_OH) |
|  | MVNHFIAEFK | 237 | 246 | 1 | CpdA (NH_4_OH) |
|  | TLSSSTQASIEIDSLYEGIDFYTSITR | 273 | 299 | 3 | CpdA (NH_4_OH) |
|  | ARFEELNADLFR | 300 | 311 | 1 | CpdA (NH_4_OH) |
|  | FEELNADLFR | 302 | 311 | 1 | CpdA (NH_4_OH) |
|  | SQIHDIVLVGGSTR | 329 | 342 | 1 | CpdA (NH_4_OH) |
|  | LLQDFFNGK | 349 | 357 | 1 | CpdA (NH_4_OH) |
|  | SINPDEAVAYGAAVQAAILSGDK | 362 | 384 | 1 | CpdA (NH_4_OH) |
|  | SENVQDLLLLDVTPLSLGIETAGGVMTVLIK | 385 | 415 | 1 | CpdA (NH_4_OH) |
|  | Q<Pyr>TQTFTTYSDNQPGVLIQVYEGER | 424 | 447 | 1 | CpdA (NH_4_OH) |
|  | QTQTFTTYSDNQPGVLIQVYEGER | 424 | 447 | 2 | CpdA (NH_4_OH) |
|  | GVPQIEVTFDIDANGILNVSAVDK | 470 | 493 | 6 | CpdA (NH_4_OH) |
|  | LSKEDIER | 510 | 517 | 1 | CpdA (NH_4_OH) |
|  | NSLESYAFNM<Mox>K | 540 | 550 | 1 | CpdA (NH_4_OH) |
|  | NSLESYAFNMK | 540 | 550 | 1 | CpdA (NH_4_OH) |
|  | NQTAEKEEFEHQQK | 584 | 597 | 2 | CpdA (NH_4_OH) |
| Heat shock cognate 71 kDa protein (HSP7C_HUMAN) | NQVAM<Mox>NPTNTVFDAK | 57 | 71 | 1 | NI (FLAG) |
|  | NQVAMNPTNTVFDAK | 57 | 71 | 3 | NI (FLAG) |
|  | RFDDAVVQSDM<Mox>K | 77 | 88 | 1 | NI (FLAG) |
|  | RFDDAVVQSDMK | 77 | 88 | 1 | NI (FLAG) |
|  | FDDAVVQSDM<Mox>K | 78 | 88 | 1 | NI (FLAG) |
|  | FDDAVVQSDMK | 78 | 88 | 1 | NI (FLAG) |
|  | HWPFM<Mox>VVNDAGRPK | 89 | 102 | 1 | NI (FLAG) |
|  | HWPFMVVNDAGR | 89 | 100 | 1 | NI (FLAG) |
|  | HWPFMVVNDAGRPK | 89 | 102 | 1 | NI (FLAG) |
|  | VQVEYKGETK | 103 | 112 | 1 | NI (FLAG) |
|  | SFYPEEVSSM<Mox>VLTK | 113 | 126 | 1 | NI (FLAG) |
|  | SFYPEEVSSMVLTK | 113 | 126 | 2 | NI (FLAG) |
|  | MKEIAEAYLGK | 127 | 137 | 2 | NI (FLAG) |
|  | EIAEAYLGK | 129 | 137 | 1 | NI (FLAG) |
|  | TVTNAVVTVPAYFNDSQR | 138 | 155 | 3 | NI (FLAG) |
|  | DAGTIAGLNVLR | 160 | 171 | 2 | NI (FLAG) |
|  | NVLIFDLGGGTFDVSILTIEDGIFEVK | 194 | 220 | 1 | NI (FLAG) |
|  | STAGDTHLGGEDFDNR | 221 | 236 | 2 | NI (FLAG) |
|  | M<Mox>VNHFIAEFK | 237 | 246 | 1 | NI (FLAG) |
|  | MVNHFIAEFK | 237 | 246 | 1 | NI (FLAG) |
|  | ARFEELNADLFR | 300 | 311 | 1 | NI (FLAG) |
|  | FEELNADLFR | 302 | 311 | 1 | NI (FLAG) |
|  | LDKSQIHDIVLVGGSTR | 326 | 342 | 1 | NI (FLAG) |
|  | SQIHDIVLVGGSTR | 329 | 342 | 2 | NI (FLAG) |
|  | LLQDFFNGK | 349 | 357 | 1 | NI (FLAG) |
|  | SINPDEAVAYGAAVQAAILSGDK | 362 | 384 | 1 | NI (FLAG) |
|  | Q<Pyr>TQTFTTYSDNQPGVLIQVYEGER | 424 | 447 | 1 | NI (FLAG) |
|  | QTQTFTTYSDNQPGVLIQVYEGER | 424 | 447 | 1 | NI (FLAG) |
|  | NSLESYAFNM<Mox>K | 540 | 550 | 1 | NI (FLAG) |
|  | NSLESYAFNMK | 540 | 550 | 2 | NI (FLAG) |
|  | NQTAEKEEFEHQQK | 584 | 597 | 2 | NI (FLAG) |
|  | LYQSAGGMPGGMPGGFPGGGAPPSGGASSGPTIEEVD | 610 | 646 | 1 | NI (FLAG) |
| Heat shock cognate 71 kDa protein (HSP7C_HUMAN) | NQVAM<Mox>NPTNTVFDAK | 57 | 71 | 1 | NI (NH_4_OH) |
|  | NQVAMNPTNTVFDAK | 57 | 71 | 1 | NI (NH_4_OH)) |
|  | RFDDAVVQSDMK | 77 | 88 | 1 | NI (NH_4_OH) |
|  | FDDAVVQSDMK | 78 | 88 | 1 | NI (NH_4_OH) |
|  | HWPFMVVNDAGRPK | 89 | 102 | 1 | NI (NH_4_OH) |
|  | VQVEYKGETK | 103 | 112 | 1 | NI (NH_4_OH) |
|  | SFYPEEVSSMVLTK | 113 | 126 | 2 | NI (NH_4_OH) |
|  | MKEIAEAYLGK | 127 | 137 | 2 | NI (NH_4_OH) |
|  | EIAEAYLGK | 129 | 137 | 1 | NI (NH_4_OH)) |
|  | TVTNAVVTVPAYFNDSQR | 138 | 155 | 2 | NI (NH_4_OH) |
|  | DAGTIAGLNVLR | 160 | 171 | 1 | NI (NH_4_OH) |
|  | NVLIFDLGGGTFDVSILTIEDGIFEVK | 194 | 220 | 1 | NI (NH_4_OH) |
|  | STAGDTHLGGEDFDNR | 221 | 236 | 2 | NI (NH_4_OH) |
|  | MVNHFIAEFK | 237 | 246 | 1 | NI (NH_4_OH) |
|  | TLSSSTQASIEIDSLYEGIDFYTSITR | 273 | 299 | 1 | NI (NH_4_OH) |
|  | ARFEELNADLFR | 300 | 311 | 1 | NI (NH_4_OH)) |
|  | FEELNADLFR | 302 | 311 | 1 | NI (NH_4_OH) |
|  | SQIHDIVLVGGSTR | 329 | 342 | 1 | NI (NH_4_OH) |
|  | LLQDFFNGK | 349 | 357 | 1 | NI (NH_4_OH) |
|  | SINPDEAVAYGAAVQAAILSGDK | 362 | 384 | 2 | NI (NH_4_OH) |
|  | Q<Pyr>TQTFTTYSDNQPGVLIQVYEGER | 424 | 447 | 1 | NI (NH_4_OH) |
|  | QTQTFTTYSDNQPGVLIQVYEGER | 424 | 447 | 2 | NI (NH_4_OH) |
|  | GVPQIEVTFDIDANGILNVSAVDK | 470 | 493 | 5 | NI (NH_4_OH)) |
|  | NSLESYAFNM<Mox>K | 540 | 550 | 1 | NI (NH_4_OH) |
|  | NSLESYAFNMK | 540 | 550 | 1 | NI (NH_4_OH) |
|  | NQTAEKEEFEHQQK | 584 | 597 | 2 | NI (NH_4_OH) |
| Heat shock protein HSP 90-beta (HS90B_HUMAN) | ELISNASDALDK | 42 | 53 | 1 | CpdA (FLAG) |
|  | YESLTDPSK | 56 | 64 | 1 | CpdA (FLAG) |
|  | ADLINNLGTIAK | 96 | 107 | 1 | CpdA (FLAG) |
|  | EDQTEYLEER | 187 | 196 | 1 | CpdA (FLAG) |
|  | YIDQEELNK | 276 | 284 | 1 | CpdA (FLAG) |
|  | NPDDITQEEYGEFYK | 292 | 306 | 1 | CpdA (FLAG) |
|  | SIYYITGESK | 482 | 491 | 1 | CpdA (FLAG) |
|  | EQVANSAFVER | 492 | 502 | 1 | CpdA (FLAG) |
| Heat shock protein HSP 90-beta (HS90B_HUMAN) | ELISNASDALDK | 42 | 53 | 1 | NI (FLAG) |
|  | ADLINNLGTIAK | 96 | 107 | 1 | NI (FLAG) |
|  | EDQTEYLEER | 187 | 196 | 1 | NI (FLAG) |
|  | YIDQEELNK | 276 | 284 | 1 | NI (FLAG) |
|  | NPDDITQEEYGEFYK | 292 | 306 | 1 | NI (FLAG) |
|  | SIYYITGESK | 482 | 491 | 1 | NI (FLAG) |
|  | EQVANSAFVER | 492 | 502 | 1 | NI (FLAG) |
| Heat shock protein HSP 90-beta (HS90B_HUMAN) | ADLINNLGTIAK | 96 | 107 | 1 | NI (NH_4_OH) |
|  | DLVVLLFETALLSSGFSLEDPQTHSNR | 653 | 679 | 2 | NI (NH_4_OH)) |
| Heat shock protein HSP 90-alpha (HS90A_HUMAN) | TLTIVDTGIGMTK | 88 | 100 | 1 | CpdA (FLAG) |
| Heat shock protein HSP 90-alpha (HS90A_HUMAN) | ADLINNLGTIAK | 101 | 112 | 1 | CpdA (NH_4_OH) |
