# Supplementary material for: Compound A, a Selective Glucocorticoid Receptor Modulator, Enhances Heat Shock Protein Hsp70 Gene Promoter Activation
Source: PLoS One. 2013 Jul 30;8(7):e69115. doi: 10.1371/journal.pone.0069115 (PMC3728325; doi:10.1371/journal.pone.0069115)
Supplement: Materials and Methods S1 — All materials and methods, as described in the manuscript are also valid for the supporting information figures S1-S10 and tables S1 and S2. Additional materials en methods to understand the supporting information figures and tables are added as a supporting file. (DOCX) [file pone.0069115.s013.docx]

MATERIALS & METHODS S1

All materials and methods, as described in the manuscript are also valid for the supporting information figures S1-S10 and tables S1 and S2. Additional materials en methods to understand the supporting information figures and tables are added below.

**Cell culture & reagents**

Human breast cancer MCF7 cells were obtained from Dr. H. Rochefort (Unité d’Endocrinologi Cellulaire et Moleculaire, Montpellier, France) [66]. These cells were cultured in DMEM supplemented with 5% fetal calf serum, 100 U/ml penicillin, 0.1 mg/ml streptomycin, glutamine 2mM, Na-pyruvate 1mM, non-essential amino acids and 10µg/ml insulin (Gibco; life technologies). This cell line was grown at 37°C under 5% CO_2_.

cDNA array analysis

MCF7 cells were treated with solvent or CpdA (10 µM) for 8h. Total RNA was isolated and reverse transcribed. cDNA array analysis was carried out on Human Stress and Toxicity Pathway Finder GEarray Q series membranes according to the instructions of the manufacturer (SuperArray Simplicity GEArray^TM^; SABiosciences). Total RNA was extracted using TRIzol Reagent (Invitrogen, Life Technologies) and was used as a template for [α-^33^P]-cDNA probe synthesis using GEarray labelling kit (SuperArray) and [α-^33^P]-dCTP (Amersham Pharmacia Biotech). Visualization of radioactive bands was carried out by a Phospho-Imager and scanned using a bioimaging analyser (Personal Molecular Imager FX BioRad). Densitometric analysis of autoradiograms was performed using Quantity One (BioRad). Signals of the displayed genes were normalized to the signal derived from the housekeeping control genes GAPDH, β-actin, cyclophilin A and ribosomal protein L13a on the same membrane. The resulting data for relative mRNA expression were presented as ‘fold induction’.
